# Supplementary material for: Severe COVID-19 anxiety among adults in the UK: protocol for a cohort study and nested feasibility trial of modified cognitive–behavioural therapy for health anxiety
Source: BMJ Open. 2022 Sep 7;12(9):e059321. doi: 10.1136/bmjopen-2021-059321 (PMC9453423; doi:10.1136/bmjopen-2021-059321)
Supplement: Supplementary data [file bmjopen-2021-059321supp001.pdf]

Imperial College London

Chief Investigator: Prof. Mike Crawford
Division of Psychiatry, Imperial College London
7th Floor Commonwealth Building, Du Cane Road
London. W12 0NN

Consent Form for the COVID Anxiety Project Separate consent for Clinical Trial of CBT for Health Anxiety

This consent form should be completed after reading the COVID Anxiety Project: Clinical Trial Information Sheet

Please sign your initials in each box to confirm the statements

I confirm that I have read and understand the information sheet for the above study. I have had the opportunity to ask questions which have been answered fully	
I understand that by taking part in the trial, I have an equal chance of being offered therapy or no therapy. If I allocated to the group that receives therapy, I will be asked to take part in a series of one-to-one telephone sessions of cognitive behavioural therapy.	
I understand that my participation is voluntary and that I am free to withdraw at any time without giving any reason, without my medical care or legal rights being affected.	
I agree to Imperial College keeping my data and using it for the purpose of this research. I understand that the responses that I give will be anonymised and kept for 10 years.	
I understand that therapy sessions will be audio recorded so that a senior member of the research team may listen to the session and provide feedback to the therapist. The recordings wil be deleted at the end of the study.	
I consent to take part in the above study.	

Please write your full name, today's date and add your signature below

____________________ Name　__________ Date　____________________ Signature

Crawford MJ, *et al. BMJ Open* 2022; 12:e059321. doi: 10.1136/bmjopen-2021-059321
