# Supplementary material for: Severe COVID-19 anxiety among adults in the UK: protocol for a cohort study and nested feasibility trial of modified cognitive–behavioural therapy for health anxiety
Source: BMJ Open. 2022 Sep 7;12(9):e059321. doi: 10.1136/bmjopen-2021-059321 (PMC9453423; doi:10.1136/bmjopen-2021-059321)
Supplement: Supplementary data [file bmjopen-2021-059321supp002.pdf]

BMJ Publishing Group Limited (BMJ) disclaims all liability and responsibility arising from any reliance placed on this supplemental material which has been supplied by the author(s)

Imperial College London

Chief Investigator: Prof. Mike Crawford
Division of Psychiatry, Imperial College London
7th Floor Commonwealth Building, Du Cane Road
London. W12 0NN

Consent Form for the COVID Anxiety Project

This consent form should be completed after reading the COVID Anxiety Project Information Sheet version 1.0, date 09.01.2021

Please sign your initials in each box to confirm the statements

Statement	Initials
I am 18 years or older.	
I have read and understand the information sheet for the COVID Anxiety Project. I have had the opportunity to consider the information, ask questions and any questions were answered satisfactorily.	
I understand that my participation is voluntary and that I am free to withdraw at any time without giving any reason, without my medical care or legal rights being affected.	
I agree for my data to be recorded and stored on the Qualtrics server in compliance with the General Data Proection Regulations (GDPR). I agree to Imperial College keeping my data and using it for the purpose of this research. I understand that the responses that I give will be anonymised and kept for 10 years.	
I understand that my contact details will be kept by Imperial College until then end of the project and then deleted.	
I understand that I will be offered a £30 voucher when I have completed the six month questionnaire.	
I agree to take part in the COVID Anxiety Project.	

Please write your full name, today's date and add your signature below

______________________ ______________ ______________________
Name of individual Date Signature

Crawford MJ, *et al. BMJ Open* 2022; 12:e059321. doi: 10.1136/bmjopen-2021-059321

Crawford MJ, *et al. BMJ Open* 2022; 12:e059321. doi: 10.1136/bmjopen-2021-059321
