# Supplementary material for: Severe COVID-19 anxiety among adults in the UK: protocol for a cohort study and nested feasibility trial of modified cognitive–behavioural therapy for health anxiety
Source: BMJ Open. 2022 Sep 7;12(9):e059321. doi: 10.1136/bmjopen-2021-059321 (PMC9453423; doi:10.1136/bmjopen-2021-059321)
Supplement: Author's manuscript [file bmjopen-2021-059321.draft_revisions.pdf]

BMJ Open is committed to open peer review. As part of this commitment we make the peer review history of every article we publish publicly available.

When an article is published we post the peer reviewers' comments and the authors' responses online. We also post the versions of the paper that were used during peer review. These are the versions that the peer review comments apply to.

The versions of the paper that follow are the versions that were submitted during the peer review process. They are not the versions of record or the final published versions. They should not be cited or distributed as the published version of this manuscript.

BMJ Open is an open access journal and the full, final, typeset and author-corrected version of record of the manuscript is available on our site with no access controls, subscription charges or pay-per-view fees (http://bmjopen.bmj.com).

If you have any questions on BMJ Open's open peer review process please email info.bmjopen@bmj.com

BMJ Open

Severe COVID anxiety among adults in the United Kingdom: protocol for a cohort study and nested feasibility trial of modified Cognitive Behaviour Therapy for Health Anxiety.

Journal:	*BMJ Open*
Manuscript ID	bmjopen-2021-059321
Article Type:	Protocol
Date Submitted by the Author:	18-Nov-2021
Complete List of Authors:	Crawford, Mike; Imperial College London, Psychological Medicine<br>Leeson, Verity; Imperial College London<br>McQuaid, Aisling; Imperial College London<br>Samuel, Oluwaseun; Imperial College London<br>King, Jacob D; Imperial College London<br>Di Simplicio, Martina; Imperial College London<br>Tyrer, Peter; Imperial College London, Psychological medicine<br>Tyrer, Helen; Imperial College London<br>Watt, Richard; University College London, Epidemiology and Public Health; King's College London, King's College Dental Institute<br>Barnicot, Kirsten; City University of London
Keywords:	COVID-19, Anxiety disorders < PSYCHIATRY, Clinical trials < THERAPEUTICS

SCHOLARONE™
Manuscripts

Severe COVID anxiety among adults in the United Kingdom: protocol for a cohort study and nested feasibility trial of modified Cognitive Behaviour Therapy for Health Anxiety.

Mike J Crawford[1], Verity C Leeson[1], Aisling McQuaid[1], Oluwaseun Samuel[1], Jacob D King[1], Martina Di Simplicio[1], Peter Tyrer[1], Helen Tyrer[1], Richard G Watt[2], Kirsten Barnicot[3].

ABSTRACT

Introduction

Some people are so anxious about COVID that it impairs their functioning. However, little is known about the course of severe COVID anxiety or what can be done to help people who experience it.

Methods and analysis

Cohort study with a nested feasibility trial with follow-up at three and six months. We recruited people to the cohort study who were aged 18 and over, lived in the United Kingdom and had severe COVID anxiety (indicated by a score of nine or more on the Coronavirus Anxiety Scale). To take part in the nested feasibility trial, participants also had to have a score of 20 or more on the Short Health Anxiety Inventory. We excluded people from the trial if they had had COVID-19 within the previous four weeks, if they were currently self-isolating or if they were already receiving psychological treatment.

We publicised the study nationally through adverts, social media and posts on chat boards. We also recruited participants via clinicians working in primary and secondary care NHS services in London. All those in the active arm will be offered five to ten sessions of remotely delivered modified Cognitive Behaviour Therapy for Health Anxiety (CBT-HA). We will examine the proportion of participants who remain above threshold on the Coronavirus Anxiety Scale at three and six months and factors that influence levels of COVID anxiety over six months using mixed-effects logistic regression. The key feasibility metrics for the nested trial are the level of uptake of CBT-HA and the rate of follow-up.

Ethics and dissemination

For peer review only - http://bmjopen.bmj.com/site/about/guidelines.xhtml

Approved by Leicester Central Research Ethics Committee (reference: 20/EM/0238). The results of the study will be published in peer-reviewed scientific journals.

Trial registration: International Standard Randomised Control Trial Number Register - ISRCTN14973494

Strengths of the study

- The first study to explore the feasibility of CBT-HA for people with severe COVID anxiety.
- Use of remote methods for data collection and delivering the intervention, which enable participation by those who are unable to attend face-to-face meetings.

Limitations of the study

- The study design means that we will not be able to estimate the prevalence of severe COVID anxiety in the general population.
- Reliance on remote methods for recruitment and follow-up may impact on the follow up rate.

INTRODUCTION

The COVID-19 pandemic is having a major impact on mental health throughout the world.(1, 2) In the UK, there was a marked increase in the proportion of people experiencing anxiety after the first wave of the pandemic (3-5), a trend which has also been seen in many other countries.(6) Fear is an appropriate response to a new infectious disease like COVID-19 that threatens health, lives and livelihoods. At the start of the pandemic it was unclear how many people were likely to become infected, what the case fatality rate was and to what extent people who survived the initial illness would go on to experience long-term negative effects. Under these circumstances, fearfulness and anxiety are to be expected. Indeed, research conducted in the early phase of the pandemic found that fear of COVID-19 can be

For peer review only - http://bmjopen.bmj.com/site/about/guidelines.xhtml

adaptive, resulting in greater adherence to public health measures aimed at limiting the spread of the virus.(7, 8) However, as the pandemic has progressed it has become clear that some people have become so anxious about COVID that it is impacting on their mental health and social functioning. People with severe COVID anxiety are more likely to report harmful use of alcohol and drugs, hopelessness and suicidal thoughts.(9, 10)

Health anxiety, in which people become overwhelmed by fears of becoming ill,(11) was a major contributor to poor mental health during previous pandemics.(12-14) Emerging evidence from public surveys in Europe, Asia and North America have shown it is also contributing to poor mental health during the COVID-19 pandemic.(15-17) People with health anxiety may repeatedly search the internet and other media for information about health conditions, leading to mental distress and impaired functioning.(18, 19) People with health anxiety may misinterpret bodily sensations as symptoms of a physical illness and seek reassurance from others.(20) However, symptom monitoring and reassurance seeking may increase the amount of time that people think about being unwell and increase their level of anxiety.(11)

During the COVID pandemic people may be at greater risk of health anxiety because of widespread media coverage, misinformation about COVID and uncertainty about the course of the pandemic.(21) In addition to this people are being actively encouraged to stay alert for signs of infection and get tested for COVID-19 as soon as possible if even mild signs of infection occur. This approach is an important public health strategy for limiting the transmission of COVID,(22) but it may also increase the likelihood of people who are predisposed to being anxious about their health becoming severely anxious to the extent that reduces their quality of life and impacts on their ability to function.(13)

The link between health anxiety and poor mental health seenin previous pandemics, is important because health anxiety is treatable. Cognitive Behavioural Therapy for Health Anxiety (CBT-HA) leads to long-term reductions in levels of health anxiety, generalised anxiety and depression.(23, 24) In recent years the intervention has been successfully modified to enable it to be delivered remotely.(25) As social distancing measures are relaxed and people are able to resume social and occupational roles, it is essential to

For peer review only - http://bmjopen.bmj.com/site/about/guidelines.xhtml

understand the course and consequences of severe COVID anxiety and examine interventions that could improve people's mental health and functioning.

However, at this stage, we do not know the course of COVID anxiety or the contribution that health anxiety plays in maintaining it. While CBT-HA is effective under normal circumstances, we need to find out if it is an appropriate intervention to offer during a global pandemic, when people continue to be asked to be vigilant in spotting the signs of infection in an effort to prevent transmission of the virus.

For peer review only

Objectives

This study aims to fill current gaps in knowledge and contribute to the development of a more effective response to health anxiety during pandemics. The study objectives are to;
1) Assess the impact of severe COVID anxiety on people's social functioning and health-related quality of life,
2) Examine the course of severe COVID anxiety over a six-month period,
3) Identify factors that influence the course of severe COVID anxiety, and
4) Test the feasibility of a randomised controlled trial of CBT-HA for people with severe COVID anxiety and health anxiety aimed at improving mental health and social functioning.

METHODS AND ANALYSIS

The COVID Anxiety Project is a cohort study with a nested feasibility trial. People taking part in the study will be followed up for six months. The trial is a researcher-blind, parallel-arm, randomised controlled feasibility trial, which is compliant with Standard Protocol Items: Recommendations for Interventional Trials guidelines.(26)

Study population and sample

Study participants were recruited from the general public in the United Kingdom and from those in contact with primary care and secondary care mental health services in London. We aimed to recruit people who self-identified as being anxious about COVID-19 using a broad range of methods including social media sites (Facebook, Reddit, Instagram and Twitter) and mental health charities (MQ research and Anxiety UK). We publicised the study via 19 primary care practices in north and west London and through colleagues working in

For peer review only - http://bmjopen.bmj.com/site/about/guidelines.xhtml

secondary care mental health services in Central and North West London NHS Foundation Trust, City and East London NHS Foundation Trust and West London NHS Trust.

To be eligible to take part in the cohort study, potential participants had to be aged 18 or over, live in the United Kingdom and score nine or more on the Coronavirus Anxiety Scale.(9) We used a threshold of nine or more on the CAS because this identifies those with moderate or severe functional impairment with 90% sensitivity, 85% specificity, and a false positive rate of 15%.(9)

We excluded people who had a current or previous diagnosis of a psychotic mental disorder. To take part in the nested feasibility trial, participants also had to have a score of 20 or more on the Short Health Anxiety Inventory (SHAI).(27) We excluded people from the feasibility trial if, at the time of the baseline assessment, they:

- had had COVID-19 in the prior four weeks (defined as a positive antigen test or a diagnosis by a clinician),
- were self-isolating on the advice of a doctor or the NHS Test and Trace service; or
- were already receiving psychological treatment for any condition.

The cohort study will be made up of all study participants with the exception of those offered CBT-HA as part of the feasibility trial.

Recruitment and follow-up

Participant flow through the study is presented in figure 1. We posted adverts on social media sites and websites of mental health charities and asked staff working in primary and secondary care NHS services to direct potential participants to a dedicated study website that was hosted by Qualtrics (www.qualtrics.com). The site included a copy of the Participant Information Sheet. Potential participants were asked to sign and date an online consent form, confirm their eligibility to take part in the study, and complete the Coronavirus Anxiety Scale. The survey was designed so that it could only be completed once from any one IP address.(28) Those who scored nine or more on the scale and met other eligibility criteria were invited to complete the baseline survey. Those who were not eligible for the study were directed to a webpage which listed sources of mental health support.

We identified potential participants for the feasibility trial from their responses to the baseline survey. We aimed to recruit participants according to the capacity of study therapists to deliver CBT-HA to those in the active arm of the trial. When therapists had capacity to work with more people, we emailed those who recently joined the cohort study

For peer review only - http://bmjopen.bmj.com/site/about/guidelines.xhtml

and met the additional eligibility criteria a copy of an additional Participant Information Sheet and directed them to an online consent form. A researcher contacted these participants by telephone to answer any queries they had about the study, confirm that they met study eligibility criteria, support them to complete the online consent form and counter sign it. The researcher then contacted the trial manager who randomised the participant. Any participant who was approached to take part in the randomised trial and was ineligible or declined to take part, remained in the cohort study and will be asked to complete subsequent follow-up surveys.

Following attempts by what appeared to be automated survey-takers or 'bots' to take part in the study, we added a CAPTCHA question and a 'trap' question.(28, 29) The 'trap' question provided a range of valid and non-valid but plausible options for how the potential participant had heard about the study, with only those providing a valid response being invited to complete the baseline survey.

Between February 2020 and September 2021, we recruited 303 participants to the study. Of these, 127 (41.9%) met inclusion criteria for the feasibility trial. Among 108 participants offered a place on the trial 40 (37.0%) accepted and were randomised. The total sample for the cohort study is 283, comprising 176 who were ineligible for the trial, 18 who were eligible but not offered a place due to limited capacity of therapists to take on new patients, 68 who were offered a place on the trial but declined it and 20 people who were randomised to treatment as usual.

All participants in the cohort study and the feasibility trial will be sent an automated email to ask them to complete the three- and six-month follow-up survey. Those who have not responded after one week will be sent reminders. Trial participants who do not complete follow-up interviews will also be contacted by researchers by email (or telephone if contact number was available) with requests to complete the follow-up surveys.

Randomisation and blinding

We generated a randomisation list using the independent web-based service 'sealed envelope' (https://www.sealedenvelope.com/simple-randomiser/v1/lists). We stratified according to score on the short Health Anxiety Inventory (≤24 and ≥25) and the Dependent Personality Questionnaire (≤11 and ≥12) using a ratio of CBT-HA to control treatment of 1:1. We included the Dependent Personality Questionnaire as a stratification variable because

For peer review only - http://bmjopen.bmj.com/site/about/guidelines.xhtml

previous research has demonstrated that the presence of dependent personality traits may influence the uptake and impact of CBT-HA.(30)

Throughout the study, the randomisation list will be encrypted and stored electronically by the trial manager. At the end of the study the randomisation list will be unencrypted and placed in the Trial Master File. The trial manager generated the allocation of each new trial participant by allocating them to the next treatment arm in the predetermined list. The participant was informed of their allocation by telephone or email, and the first therapy session scheduled for those allocated to CBT-HA. Researchers will have very little contact with participants other than to encourage them to complete follow up surveys. In the unlikely event that researchers speak to a study participant after they have been randomised, they start by reminding them of the need to make sure they are not aware of whether the participant was offered CBT-HA or control treatment.

Assessments

The timing and sequence of all assessments are summarised in table 1.

Screening assessment

To take part in the cohort study, potential participants had to score nine or more on the Coronavirus Anxiety Scale (CAS).(9, 10) Developed by Sherman Lee in the USA, the scale was designed to provide a brief and reliable measure of the level of COVID-related anxiety that people experience. It comprises five questions on the frequency of anxious thoughts, somatic symptoms and sleep disturbance triggered by reading, thinking or hearing about COVID.

Baseline assessment and covariates

At baseline we collected self-reported data on demographic factors (age, gender, ethnicity, household composition, occupational status), physical health (current medical conditions), and exposure to COVID (whether they had had COVID, whether they had been admitted to hospital with COVID). We asked participants whether they live with or care for someone that might get seriously ill if infected with COVID because of their health or age and whether someone in their family or a close friend had been in hospital with COVID.

We also asked participants questions about behaviours intended to reduce the risk of exposure to COVID. These questions were developed with the help of the members of the Lived Experience Advisory Panel and covered five behaviours which people had used or heard of others using in an effort to avoid catching COVID: staying at home, avoiding shops,

For peer review only - http://bmjopen.bmj.com/site/about/guidelines.xhtml

washing or discarding letters and parcels, increased handwashing and increased washing of clothes. People who lived with school age children were also asked whether they had stopped them attending school because of concerns about COVID.

We assessed self-reported functioning using the Work and Social Adjustment scale (WSAS)(31) and health-related quality of life using the EQ-5D-3L.(32) We assessed mental health using the Patient Health Questionnaire-9,(33) Generalised Anxiety Disorder 7-item scale,(34) Obsessive -Compulsive Inventory -Revised,(35) the short form of the Health Anxiety Inventory (SHAI), the Standardised Assessment of Personality – Abbreviated Scale,(36) the Dependent Personality Questionnaire,(37) use of alcohol (AUDIT-C) (38) and a single-question screening test for drug use. Finally, we asked participants to indicate admissions to hospital, contacts with primary and secondary care services using items from the Adult Service User Schedule.(39)

Follow-up surveys

All those taking part in the cohort study or the feasibility trial are asked to complete follow-up surveys three and six months after the baseline survey. The content of these surveys was similar to the baseline survey, except that we did not repeat the personality assessments (SAPAS and DPQ), and we added a question at six months on whether people had received a COVID-19 vaccination (see table 1).

In recognition of their time and support, all those who completed a baseline interview were sent a £10 gift voucher and a further £20 voucher will be offered to those that complete the six-month follow-up interview.

Serious adverse events

Data on Serious Adverse Event (SAE) including hospital admission are recorded at baseline, and three and six month follow-up. Therapists will also be regularly reminded that they should report all serious adverse events to the clinical trial team. SAEs will be recorded on a non-CTIMP SAE form and forwarded within 24 hours to the Chief Investigator and sponsor. The Research Ethics Committee will be notified within 15 days of the Chief Investigator becoming aware of any SAE where the event resulted from the administration of any of the research procedures.

Interventions

All those who agree to take part in the feasibility trial will be sent a self-help booklet developed by staff at Central and North West London NHS Foundation Trust that is derived

For peer review only - http://bmjopen.bmj.com/site/about/guidelines.xhtml

from the work of Russ Harris on Acceptance and Commitment Therapy,(40) and aims to help people cope with the COVID crisis by building resilience, identifying and implementing healthy coping strategies and general advice on health and wellbeing.(41) All participants will be able access treatment as usual, which includes access to NHS primary care services and referral on to specialist services if required.

All those in the active arm of the feasibility trial will be offered five to ten sessions of modified Cognitive Behavioural Therapy for Health Anxiety (CBT-HA) based on a published treatment manual.(42)Therapists will start by taking a detailed history of the person's thoughts and fears about COVID and exploring their beliefs about the impact of COVID and their ability to cope, in order to develop a formulation. Therapists will seek to identify behaviours such as symptom monitoring and reassurance seeking that could be maintaining the person's anxiety. They will then use Socratic dialogue, diary keeping and behavioural experiments to help participants recognise the links between their thoughts and behaviour and explore ways to reduce their anxiety.

All CBT-HA sessions will be delivered by phone or videoconferencing, in accordance with the participant's preference. Sessions will generally last between 30 and 50 minutes and be offered on a weekly or fortnightly basis. Therapists will consider delaying their final session in order to give people an opportunity to use the techniques and skills they have learned and reinforce the changes they have made. Sessions will be delivered in keeping with a published treatment manual but modified to meet the difficulties that people in the study are experiencing. Sessions will be supplemented by booklets summarising the causes of health anxiety and how patients can begin to overcome their fears.

Each therapist will record the number and length of sessions they offer participants so that we can calculate the proportion of people who are offered CBT-HA who start and complete treatment and the number of sessions that people receive.

All therapists have a degree in a health-related subject and have previous experience of delivering psychological treatments. All therapists attended a 90-minute training session by Dr Helen Tyrer and will receive fortnightly supervision sessions delivered by Dr Tyrer.

Sample size

We aimed to recruit sufficient numbers of participants to the cohort study in order to randomise 40 participants to the feasibility trial, which is a typical size for such a trial.(43) A

For peer review only - http://bmjopen.bmj.com/site/about/guidelines.xhtml

sample of 40 participants would enable us to detect study consent and intervention completion rates of 50%, with 95% confidence intervals of +/-11% and +/- 15% respectively. Assuming a rate of follow-up at six months of 75%, our cohort of 283 study participants will generate valid data on 212 people. A sample of 196 participants in the cohort study will enable us to estimate a remission rate of 15% +/- 5%, with 95% confidence.(44)

Data analysis

We will start by examining the characteristics of the study sample, including the prevalence of coexisting physical and mental health conditions, levels of disturbance in work and social functioning, quality of life, and actions taken to try to avoid catching COVID using simple descriptive statistics. For the cohort study, we will exclude those randomised to the active treatment arm of the nested feasibility trial, but include those in the control arm of the trial. We will examine the proportion who remain above threshold for severe COVID anxiety (9 or above on the CAS) and for COVID anxiety (5 or more on the CAS) at three and six months after completion of the baseline survey. We will examine factors that influence changes in scores over this period using linear regression analysis. With a condensed list of covariates we anticipate running mixed-effects linear regression models with maximum likelihood estimation, while fixing time and covariates, and utilising random effects to adjust for subject-specific heterogeneities. We will use mixed-effect logistic regression to conduct a sensitivity analysis, to examine whether factors predictive of improvement over time are also predictive of ceasing to meet the threshold for severe COVID anxiety.

We anticipate that there will be little missing data because the platform we designed for data entry requires participants to complete every item of a questionnaire before moving on to the next section. However where the number of missing items on a given measure is 20% or less, then the missing value for the items will be substituted by the individual's mean score for the remaining items on the scale. If there are more than 20% missing items in the scale the outcome measure will not be calculated for the participant at that time point.

Our criteria for determining the success of the feasibility study are: recruitment of at least 32 participants (80% of the target study sample of 40 participants), uptake of the intervention by at least 60% of participants in the active arm of the trial, and completion of follow-up interviews at six months by 75% of study participants. All data will be analysed in SPSS version 2.0 (SPSS Inc., Chicago IL) and Stata version 16.1.(45)

For peer review only - http://bmjopen.bmj.com/site/about/guidelines.xhtml

Patient and public involvement

Patients and the public contributed to the development of this proposal. Charlotte Green, who chaired the Patient & Carers Research Interest group at CNWL NHS Foundation Trust, helped develop the proposal and write the lay summary. We presented plans for the study to members of a local patient research interest group via Zoom. Members expressed strong support for the study and eight agreed to join a Lived Experience Advisory Group. Feedback from the group led to us to adding new questions on the impact of COVID anxiety. Members made suggestions for how to access potential participants, which we adopted. Members of the group also highlighted the importance of tailoring CBT-HA to the needs of individual patients, including making time for them to discuss broader concerns about their mental health and the impact of the pandemic. Members of the Lived Experience Advisory Group will help us prepare a plain English summary of study findings, which we will make available to all study participants and other members of the public through the study website.

ETHICS AND DISSEMINATION

Plans for the study were approved by Leicester Central Research Ethics Committee (reference: 20/EM/0238). The study will be conducted in accordance with the recommendations for physicians involved in research on human subjects adopted by the 18th World Medical Assembly, Helsinki 1964 and later revisions.

All research activity will be carried out after participants have given consent, and this documented on either an electronic or paper form. For the cohort study, the consent forms will be completed independently by the participant without researcher support but a member of the study team will be available to answer any questions prior to consent being given. Individual consent completed for the cohort study will be linked in Qualtrics to the questionnaires completed by the same participant at each timepoint in the cohort study to ensure that the person that gave consent is completing the assessments.

Separate consent to take part in the clinical trial will be completed by the participant during a telephone call with a member of the research team. The member of the research team that facilitates the completion of the consent process on the telephone will countersign the consent form to document that they were present.

For peer review only - http://bmjopen.bmj.com/site/about/guidelines.xhtml

Completed electronic consent forms will be downloaded from Qualtrics, printed and stored in a Master File. A copy of their completed consent form(s) will be provided to each participant. Any original paper consent forms will also be stored in the Master File. All participants are free to withdraw at any time from the cohort study or clinical trial without giving reasons and without prejudicing further treatment.

The Chief Investigator will preserve the confidentiality of participants taking part in the study and is registered under the Data Protection Act. Data from the consent forms and questionnaires, including identifiable personal data, will be stored on Qualtrics, an online survey tool. Qualtrics has ISO 27001 certification, meaning that it meets the international standard for managing confidential and sensitive data. All data transfers to Imperial College London use TLS encryption. Imperial College will be the data controller and there is a service-level agreement in place between Imperial College and Qualtrics.

Where questionnaires are administered during a telephone interview rather than online, the researcher will type the responses into Qualtrics. Other personal data that is recorded during the study (e.g. therapy notes from CBT sessions) will be typed directly onto the secure server of Imperial College London and will be identified using only the participant identifier. Qualtrics allows data erasure and all study data will be deleted from Qualtrics at the end of the study, following extraction for analysis.

Paper documentation will be minimal during this research. An option to complete paper consent form and screening questionnaires was offered but not taken up by any participant.

The results of the study will be published in peer-reviewed scientific journals. We anticipate three key papers examining baseline data, follow-up data and the results of the feasibility trial. We will work with members of our Lived Experienced Advisory Panel to prepare a lay summary of findings from the study which we will post on the study website and will email all study participants the link to this summary.

AUTHOR AFFILIATIONS

1) Professor Mike J Crawford (Corresponding author). Division of Psychiatry, Imperial College London, Commonwealth Building, Hammersmith Hospital Campus, Du Cane Road, London W12 0NN, UK. m.crawford@imperial.ac.uk

For peer review only - http://bmjopen.bmj.com/site/about/guidelines.xhtml

2) Dr Verity Leeson. Division of Psychiatry, Imperial College London, Commonwealth Building, Hammersmith Hospital Campus, Du Cane Road, London W12 0NN, UK. v.leeson@imperial.ac.uk
3) Ms Aisling McQuaid. Division of Psychiatry, Imperial College London, Commonwealth Building, Hammersmith Hospital Campus, Du Cane Road, London W12 0NN, UK. a.mc-quaid@imperial.ac.uk
4) Ms Oluwaseun Samuel. Division of Psychiatry, Imperial College London, Commonwealth Building, Hammersmith Hospital Campus, Du Cane Road, London W12 0NN, UK. o.samuel@imperial.ac.uk
5) Dr Jacob King. Division of Psychiatry, Imperial College London, Commonwealth Building, Hammersmith Hospital Campus, Du Cane Road, London W12 0NN, UK. j.king20@imperial.ac.uk
6) Dr Martina Di Simplicio. Division of Psychiatry, Imperial College London, Commonwealth Building, Hammersmith Hospital Campus, Du Cane Road, London W12 0NN, UK. m.di-simplicio@imperial.ac.uk
7) Professor Peter Tyrer. Division of Psychiatry, Imperial College London, Commonwealth Building, Hammersmith Hospital Campus, Du Cane Road, London W12 0NN, UK. p.tyrer@imperial.ac.uk
8) Dr Helen Tyrer, Division of Psychiatry, Imperial College London, Commonwealth Building, Hammersmith Hospital Campus, Du Cane Road, London W12 0NN, UK. htyrer07@gmail.com
9) Professor Richard G Watt Department of Epidemiology & Public Health, University College London, 1-19 Torrington Place, London, WC1E 7HB, UK. r.watt@ucl.ac.uk
10) Dr Kirsten Barnicot. Division of Health Services Research and Management, City, University of London, Northampton Square, London EC1V 0HB, UK. Kirsten.Barnicot@city.ac.uk

Authors' contributions

MC is the Chief Investigator who conceived the study and led the design of the study and the study protocol and preparation of this manuscript. VL helped design the study protocol, obtained study approvals and contributed to the preparation of this manuscript. AMcQ, OS and JK recruited study participants contributed to the design of the study and the study protocol and preparation of this manuscript. HT helped to develop the study intervention and contributed to the preparation of this manuscript. MdS, RW, PT and KB, contributed to the design of the study, the development of the study protocol and the preparation of this manuscript. All authors read and approved the final manuscript.

Acknowledgements

We are grateful to the members of a Combined Independent Oversight Committee which will oversee project governance, data management and review safety data (Dr John Green (chair), Professor Khalida Ismail and Mr Robert Koch). We also thank members of the lived

For peer review only - http://bmjopen.bmj.com/site/about/guidelines.xhtml

experience reference group including Manisha Ahya, Anjie Chhapia, Charlotte Green, Sandra Jayacodi, Niruben Patel and Vikas Sharma.

Funding statement

This research is supported by funding from the National Institute for Health Research (Ref: 16/157/02) and an NIHR Senior Investigator Application held by Professor Crawford (NF-SI-0515-10006). Infrastructure support for this research is being provided by the NIHR Imperial Biomedical Research Centre (BRC not necessarily those of the NIHR, the Department of Health and Social Care or the study sponsor).The sponsor of the trial is Imperial College London.

COMPETING INTERESTS STATEMENT

Dr Helen Tyrer is author of a book on Cognitive Behaviour Therapy for treating people with Health Anxiety. Other authors declare that they have no competing interests.

REFERENCES

1. World Health Organization. Mental Health Considerations During COVID-19 Outbreak Geneva: World Health Organization, 2020.
2. Thombs BD, Bonardi O, Rice DB, Boruff JT, Azar M, He C, et al. Curating evidence on mental health during COVID-19: A living systematic review. Journal of Psychosomatic Research. 2020;133:110113.
3. Pierce M, Hope H, Ford T, Hatch S, Hotopf M, John A, et al. Mental health before and during the COVID-19 pandemic: a longitudinal probability sample survey of the UK population. The lancet Psychiatry. 2020;7(10):883-92.
4. Jia R, Ayling K, Chalder T, Massey A, Broadbent E, Coupland C, et al. Mental health in the UK during the COVID-19 pandemic: cross-sectional analyses from a community cohort study. BMJ Open. 2020;10:e040620.
5. Santabarbara J, Lasheras I, Lipnicki DM, Bueno-Notivol J, Perez-Moreno M, Lopez-Anton R, et al. Prevalence of anxiety in the COVID-19 pandemic: An updated meta-analysis of community-based studies. Prog Neuropsychopharmacol Biol Psychiatry. 2021;109:110207.

For peer review only

6. Collaborators C-MD. Global prevalence and burden of depressive and anxiety disorders in 204 countries and territories in 2020 due to the COVID-19 pandemic. Lancet. 2021;398:1700-12.

7. Harper CA, Satchell LP, Fido D, Latzman RD. Functional Fear Predicts Public Health Compliance in the COVID-19 Pandemic. Int J Ment Health Addict. 2020:1-14.

8. Chen SX, Ng JCK, Hui BPH, Au AKY, Wu WCH, Lam BCP, et al. Dual impacts of coronavirus anxiety on mental health in 35 societies. Sci Rep. 2021;11:8925.

9. Lee SA. Coronavirus Anxiety Scale: A brief mental health screener for COVID-19 related anxiety. Death Stud. 2020;44:393-401.

10. Lee SA, Mathis AA, Jobe MC, Pappalardo EA. Clinically significant fear and anxiety of COVID-19: A psychometric examination of the Coronavirus Anxiety Scale. Psychiatry Res. 2020;290:113112.

11. Tyrer P, Eilenberg T, Fink P, Hedman E, Tyrer H. Health anxiety: the silent, disabling epidemic. BMJ. 2016;353:2250.

12. Wheaton MG, Abramowitz JS, Berman NC, Fabricant LE, Olatunji BO. Psychological predictors of anxiety in response to the H1N1 (swine flu) pandemic. Cognitive Therapy and Research. 2012;36:210-8.doi:10.1007/s10608-011-9353-3.

13. Asmundson GJG, Taylor S. How health anxiety influences responses to viral outbreaks like COVID-19: What all decision-makers, health authorities, and health care professionals need to know. J Anxiety Disord. 2020;71:102211.

14. Blakey SM, Abramowitz JS. Psychological Predictors of Health Anxiety in Response to the Zika Virus. J Clin Psychol Med Settings. 2017;24:270-8.

15. Jungmann SM, Witthoft M. Health anxiety, cyberchondria, and coping in the current COVID-19 pandemic: Which factors are related to coronavirus anxiety? J Anxiety Disord. 2020;73:102239.

16. Nikcevic AV, Marino C, Kolubinski DC, Leach D, Spada MM. Modelling the contribution of the Big Five personality traits, health anxiety, and COVID-19 psychological distress to generalised anxiety and depressive symptoms during the COVID-19 pandemic. J Affect Disord. 2021;279:578-84.

For peer review only

For peer review only - http://bmjopen.bmj.com/site/about/guidelines.xhtml

17. Ozdin S, Bayrak Ozdin S. Levels and predictors of anxiety, depression and health anxiety during COVID-19 pandemic in Turkish society: The importance of gender. Int J Soc Psychiatry. 2020;66:504-11.

18. Te Poel F, Baumgartner SE, Hartmann T, Tanis M. The curious case of cyberchondria: A longitudinal study on the reciprocal relationship between health anxiety and online health information seeking. J Anxiety Disord. 2016;43:32-40.

19. McMullan RD, Berle D, Arnaez S, Starcevic V. The relationships between health anxiety, online health information seeking, and cyberchondria: Systematic review and meta-analysis. J Affect Disord. 2019;245:270-8.

20. Olatunji BO, Deacon BJ, Abramowitz JS, Valentiner DP. Body vigilance in nonclinical and anxiety disorder samples: Structure, correlates, and prediction of health concerns. Behavior Therapy. 2007;38:392-401.

21. Tyrer P. COVID-19 health anxiety. World Psychiatry. 2020;19(3):307-8.

22. Girum T, Lentiro K, Geremew M, Migora B, Shewamare S. Global strategies and effectiveness for COVID-19 prevention through contact tracing, screening, quarantine, and isolation: a systematic review. Trop Med Health. 2020;48:91.

23. Tyrer P, Cooper S, Salkovskis P, Tyrer H, Crawford M, Byford S, et al. Clinical and cost-effectiveness of cognitive behaviour therapy for health anxiety in medical patients: a multicentre randomised controlled trial. Lancet. 2014;383:219-25.

24. Cooper K, Gregory JD, Walker I, Lambe S, Salkovskis PM. Cognitive Behaviour Therapy for Health Anxiety: A Systematic Review and Meta-Analysis. Behavioural and cognitive psychotherapy. 2017;45:110-23.

25. Morriss R, Patel S, Malins S, Guo B, Higton F, James M, et al. Clinical and economic outcomes of remotely delivered cognitive behaviour therapy versus treatment as usual for repeat unscheduled care users with severe health anxiety: a multicentre randomised controlled trial. BMC Med. 2019;17:16.

26. Chan AW, Tetzlaff JM, Altman DG, Laupacis A, Gotzsche PC, Krleza-Jeric K, et al. SPIRIT 2013 statement: defining standard protocol items for clinical trials. Ann Intern Med. 2013;158:200-7.

For peer review only - http://bmjopen.bmj.com/site/about/guidelines.xhtml

27. Salkovskis PM, Rimes KA, Warwick HM, Clark DM. The Health Anxiety Inventory: development and validation of scales for the measurement of health anxiety and hypochondriasis. Psychol Med. 2002;32:843-53.

28. Storozuk A, Ashley M, Delage V, Maloney E. Got Bots? Practical Recommendations to Protect Online Survey Data from Bot Attacks. The Quantitative Methods for Psychology. 2020;16:472–81.

29. Liu M, Wronski L. Trap questions in online surveys: Results from three web survey experiments. International Journal of Market Research. 2018;60:32-49.

30. Sanatinia R, Wang D, Tyrer P, Tyrer H, Crawford M, Cooper S, et al. Impact of personality status on the outcomes and cost of cognitive-behavioural therapy for health anxiety. The British journal of psychiatry : the journal of mental science. 2016;209:244-50.

31. Mundt JC, Marks IM, Shear MK, Greist JH. The Work and Social Adjustment Scale: a simple measure of impairment in functioning. British Journal of Psychiatry. 2002;180:461-4.

32. Brooks RG. EuroQoL- the current state of play. Health Policy. 1996;37:53-72.

33. Kroenke K, Spitzer RL, Williams JB. The PHQ-9: validity of a brief depression severity measure. J Gen Intern Med. 2001;16:606-13.

34. Spitzer RL, Kroenke K, Williams JB, Lowe B. A brief measure for assessing generalized anxiety disorder: the GAD-7. Arch Intern Med. 2006;166:1092-7.

35. Foa EB, Huppert JD, Leiberg S, Langner R, Kichic R, Hajcak G, et al. The Obsessive-Compulsive Inventory: development and validation of a short version. Psychol Assess. 2002;14:485-96.

36. Moran P, Leese M, Lee T, Walters P, Thornicroft G, Mann A. Standardised Assessment of Personality - Abbreviated Scale (SAPAS): preliminary validation of a brief screen for personality disorder. British Journal of Psychiatry. 2003;183:228-32.

37. Tyrer P, Morgan J, Cicchetti D. The Dependent Personality Questionnaire (DPQ): a screening instrument for dependent personality. Int J Soc Psychiatry. 2004;50(1):10-7.

38. Dawson DA, Grant BF, Stinson FS, Zhou Y. Effectiveness of the derived alcohol use disorders identification test (AUDIT-C) in screening for alcohol use disorders and risk

For peer review only

For peer review only - http://bmjopen.bmj.com/site/about/guidelines.xhtml

drinking in the US general population. Alcoholism: Clinical and Experimental Research. 2005;29:844-54.

39. Borschmann R, Barrett B, Hellier JM, Byford S, Henderson C, Rose D, et al. Joint crisis plans for people with borderline personality disorder: feasibility and outcomes in a randomised controlled trial. British Journal of Psychiatry. 2013;202:357-64.

40. Harris R. The Happiness Trap: Stop Struggling, Start Living. London: Robinson; 2008.

41. Kemp R. Face COVID. How to respond effectively to the Corona crisis. London: Central and North West London NHS Foundation Trust, 2020.

42. Tyrer H. Tackling Health Anxiety: A CBT Handbook. Cambridge: Cambridge University Press, 2013.

43. Billingham SA, Whitehead AL, Julious SA. An audit of sample sizes for pilot and feasibility trials being undertaken in the United Kingdom registered in the United Kingdom Clinical Research Network database. BMC Medical Research Methodology. 2013;13:104.

44. Dhand NK, Khatkar MS. Statulator: An online statistical calculator. Sample Size Calculator for Estimating a Single Proportion. 2014.

45. StataCorp. Stata Statistical Software: Release 17. College Station, TX: StataCorp LLC, 2021.

For peer review only

For peer review only - http://bmjopen.bmj.com/site/about/guidelines.xhtml

Table 1: Study Assessment Schedule

Assessments	Screening	Baseline	3-month follow-up	6-month follow-up
Coronavirus Anxiety Scale	X	-	X	X
Single item psychosis history	X			
Demographic and clinical data (Age, gender, ethnicity, household composition, occupational status, medical history)	-	X	-	-
Standardised Assessment of Personality – Abbreviated Scale	-	X	-	-
Dependent Personality Questionnaire	-	X	-	-
Use of alcohol and drugs (AUDIT-C and the single-question screening test for drug use)	-	X	X	X
Generalised Anxiety Disorder 7-item scale	-	X	X	X
Short Health Anxiety Inventory	-	X	X	X
Patient Health Questionnaire-9	-	X	X	X
Work and Social Adjustment Scale	-	X	X	X
Behaviours intended to reduce the risk of exposure to COVID-19	-	X	X	X
Obsessive -Compulsive Inventory	-	X	X	X
Health-related quality of life (EQ-5D-3L)	-	X	X	X
Resource use (ADSUS)	-	X	X	X
Serious Adverse Events	-	X	X	X
Vaccination status	-	-	-	X
Trial participants only: number and length of therapy sessions received	-	-	-	X

For peer review only - http://bmjopen.bmj.com/site/about/guidelines.xhtml

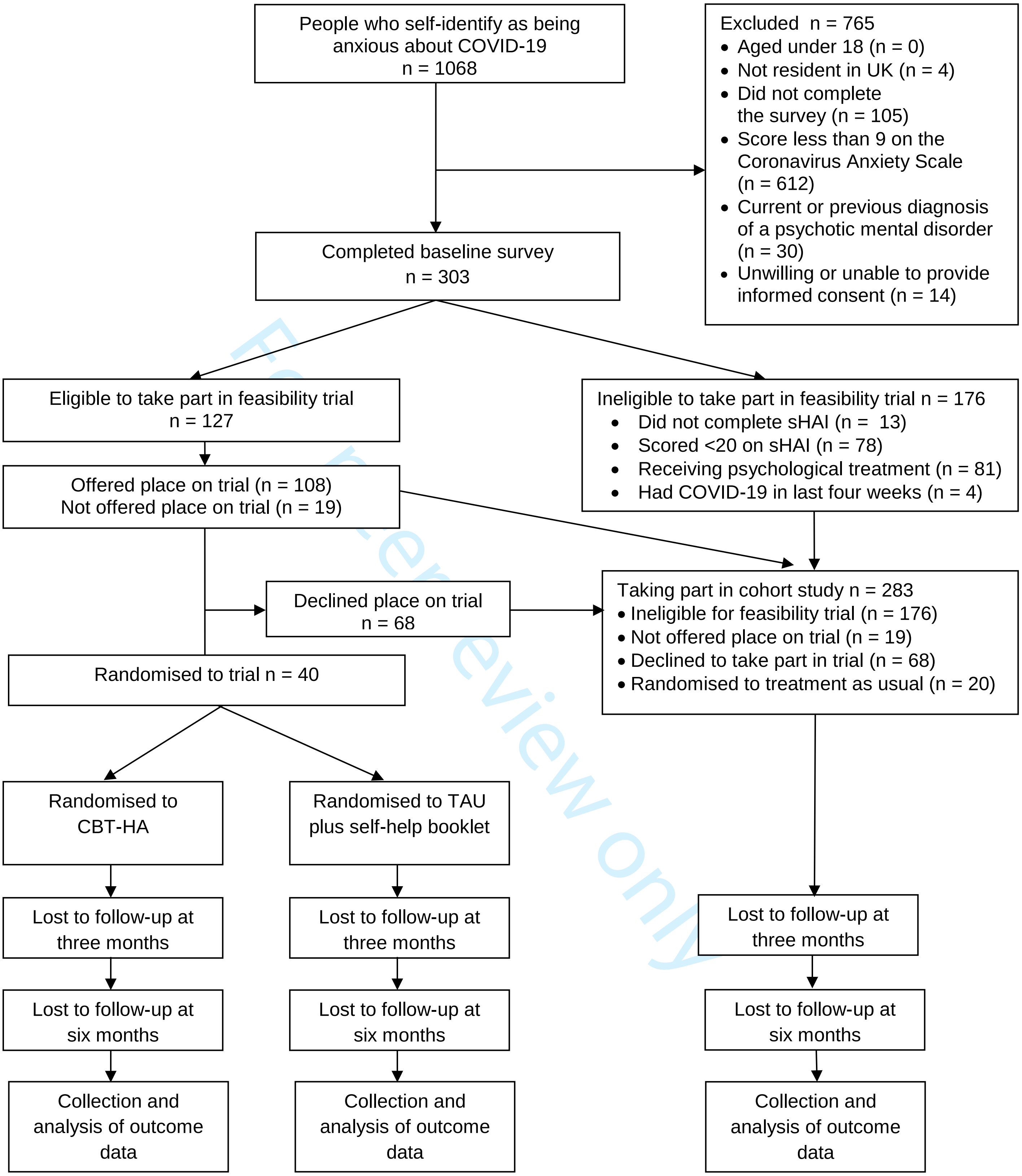

CBT-HA = Cognitive Behaviour Therapy for health anxiety. sHAI = Short for of Health Anxiety Inventory. TAU = Treatment as usual

Figure 1. Study flow diagram

For peer review only - http://bmjopen.bmj.com/site/about/guidelines.xhtml

SPIRIT

STANDARD PROTOCOL ITEMS: RECOMMENDATIONS FOR INTERVENTIONAL TRIALS

SPIRIT 2013 Checklist: Recommended items to address in a clinical trial protocol and related documents*

Section/item	Item No	Description	Page number
**Administrative information**			
Title	1	Descriptive title identifying the study design, population, interventions, and, if applicable, trial acronym	1
Trial registration	2a	Trial identifier and registry name. If not yet registered, name of intended registry	2
	2b	All items from the World Health Organization Trial Registration Data Set	throughout
Protocol version	3	Date and version identifier	
Funding	4	Sources and types of financial, material, and other support	14
Roles and responsibilities	5a	Names, affiliations, and roles of protocol contributors	14
	5b	Name and contact information for the trial sponsor	5
	5c	Role of study sponsor and funders, if any, in study design; collection, management, analysis, and interpretation of data; writing of the report; and the decision to submit the report for publication, including whether they will have ultimate authority over any of these activities	14
	5d	Composition, roles, and responsibilities of the coordinating centre, steering committee, endpoint adjudication committee, data management team, and other individuals or groups overseeing the trial, if applicable (see Item 21a for data monitoring committee)	14
**Introduction**			
Background and rationale	6a	Description of research question and justification for undertaking the trial, including summary of relevant studies (published and unpublished) examining benefits and harms for each intervention	3-4
	6b	Explanation for choice of comparators	9-10
Objectives	7	Specific objectives or hypotheses	4

For peer review only

For peer review only - http://bmjopen.bmj.com/site/about/guidelines.xhtml

Trial design	8	Description of trial design including type of trial (eg, parallel group, crossover, factorial, single group), allocation ratio, and framework (eg, superiority, equivalence, noninferiority, exploratory)	4
**Methods: Participants, interventions, and outcomes**			
Study setting	9	Description of study settings (eg, community clinic, academic hospital) and list of countries where data will be collected. Reference to where list of study sites can be obtained	5
Eligibility criteria	10	Inclusion and exclusion criteria for participants. If applicable, eligibility criteria for study centres and individuals who will perform the interventions (eg, surgeons, psychotherapists)	5
Interventions	11a	Interventions for each group with sufficient detail to allow replication, including how and when they will be administered	9-10
	11b	Criteria for discontinuing or modifying allocated interventions for a given trial participant (eg, drug dose change in response to harms, participant request, or improving/worsening disease)	Not applicable
	11c	Strategies to improve adherence to intervention protocols, and any procedures for monitoring adherence (eg, drug tablet return, laboratory tests)	Not applicable
	11d	Relevant concomitant care and interventions that are permitted or prohibited during the trial	9
Outcomes	12	Primary, secondary, and other outcomes, including the specific measurement variable (eg, systolic blood pressure), analysis metric (eg, change from baseline, final value, time to event), method of aggregation (eg, median, proportion), and time point for each outcome. Explanation of the clinical relevance of chosen efficacy and harm outcomes is strongly recommended	7-8, 11
Participant timeline	13	Time schedule of enrolment, interventions (including any run-ins and washouts), assessments, and visits for participants. A schematic diagram is highly recommended (see Figure)	20
Sample size	14	Estimated number of participants needed to achieve study objectives and how it was determined, including clinical and statistical assumptions supporting any sample size calculations	10

Recruitment	15	Strategies for achieving adequate participant enrolment to reach target sample size	5-6
**Methods: Assignment of interventions (for controlled trials)**			
Allocation:			
Sequence generation	16a	Method of generating the allocation sequence (eg, computer-generated random numbers), and list of any factors for stratification. To reduce predictability of a random sequence, details of any planned restriction (eg, blocking) should be provided in a separate document that is unavailable to those who enrol participants or assign interventions	7
Allocation concealment mechanism	16b	Mechanism of implementing the allocation sequence (eg, central telephone; sequentially numbered, opaque, sealed envelopes), describing any steps to conceal the sequence until interventions are assigned	7
Implementatio n	16c	Who will generate the allocation sequence, who will enrol participants, and who will assign participants to interventions	7
Blinding (masking)	17a	Who will be blinded after assignment to interventions (eg, trial participants, care providers, outcome assessors, data analysts), and how	6-7
	17b	If blinded, circumstances under which unblinding is permissible, and procedure for revealing a participant's allocated intervention during the trial	Not applicable
**Methods: Data collection, management, and analysis**			
Data collection methods	18a	Plans for assessment and collection of outcome, baseline, and other trial data, including any related processes to promote data quality (eg, duplicate measurements, training of assessors) and a description of study instruments (eg, questionnaires, laboratory tests) along with their reliability and validity, if known. Reference to where data collection forms can be found, if not in the protocol	8
	18b	Plans to promote participant retention and complete follow-up, including list of any outcome data to be collected for participants who discontinue or deviate from intervention protocols	8

For peer review only - http://bmjopen.bmj.com/site/about/guidelines.xhtml

Data management	19	Plans for data entry, coding, security, and storage, including any related processes to promote data quality (eg, double data entry; range checks for data values). Reference to where details of data management procedures can be found, if not in the protocol	12-13
Statistical methods	20a	Statistical methods for analysing primary and secondary outcomes. Reference to where other details of the statistical analysis plan can be found, if not in the protocol	10-11
	20b	Methods for any additional analyses (eg, subgroup and adjusted analyses)	11
	20c	Definition of analysis population relating to protocol non-adherence (eg, as randomised analysis), and any statistical methods to handle missing data (eg, multiple imputation)	10-11
**Methods: Monitoring**			
Data monitoring	21a	Composition of data monitoring committee (DMC); summary of its role and reporting structure; statement of whether it is independent from the sponsor and competing interests; and reference to where further details about its charter can be found, if not in the protocol. Alternatively, an explanation of why a DMC is not needed	14
	21b	Description of any interim analyses and stopping guidelines, including who will have access to these interim results and make the final decision to terminate the trial	Not applicable
Harms	22	Plans for collecting, assessing, reporting, and managing solicited and spontaneously reported adverse events and other unintended effects of trial interventions or trial conduct	8-9
Auditing	23	Frequency and procedures for auditing trial conduct, if any, and whether the process will be independent from investigators and the sponsor	12
**Ethics and dissemination**			
Research ethics approval	24	Plans for seeking research ethics committee/institutional review board (REC/IRB) approval	1, 11-12

For peer review only - http://bmjopen.bmj.com/site/about/guidelines.xhtml

Protocol amendments	25	Plans for communicating important protocol modifications (eg, changes to eligibility criteria, outcomes, analyses) to relevant parties (eg, investigators, REC/IRBs, trial participants, trial registries, journals, regulators)	Not applicable
Consent or assent	26a	Who will obtain informed consent or assent from potential trial participants or authorised surrogates, and how (see Item 32)	11-12
	26b	Additional consent provisions for collection and use of participant data and biological specimens in ancillary studies, if applicable	Not applicable
Confidentiality	27	How personal information about potential and enrolled participants will be collected, shared, and maintained in order to protect confidentiality before, during, and after the trial	12
Declaration of interests	28	Financial and other competing interests for principal investigators for the overall trial and each study site	14
Access to data	29	Statement of who will have access to the final trial dataset, and disclosure of contractual agreements that limit such access for investigators	14
Ancillary and post-trial care	30	Provisions, if any, for ancillary and post-trial care, and for compensation to those who suffer harm from trial participation	Not applicable
Dissemination policy	31a	Plans for investigators and sponsor to communicate trial results to participants, healthcare professionals, the public, and other relevant groups (eg, via publication, reporting in results databases, or other data sharing arrangements), including any publication restrictions	12-13
	31b	Authorship eligibility guidelines and any intended use of professional writers	Not applicable
	31c	Plans, if any, for granting public access to the full protocol, participant-level dataset, and statistical code	Not applicable
**Appendices**			
Informed consent materials	32	Model consent form and other related documentation given to participants and authorised surrogates	Available on request
Biological specimens	33	Plans for collection, laboratory evaluation, and storage of biological specimens for genetic or molecular analysis in the current trial and for future use in ancillary studies, if applicable	Not applicable

For peer review only

*It is strongly recommended that this checklist be read in conjunction with the SPIRIT 2013 Explanation & Elaboration for important clarification on the items. Amendments to the protocol should be tracked and dated. The SPIRIT checklist is copyrighted by the SPIRIT Group under the Creative Commons "Attribution-NonCommercial-NoDerivs 3.0 Unported" license.

For peer review only

Severe COVID anxiety among adults in the United Kingdom: protocol for a cohort study and nested feasibility trial of modified Cognitive Behaviour Therapy for Health Anxiety.

Journal:	*BMJ Open*
Manuscript ID	bmjopen-2021-059321.R1
Article Type:	Protocol
Date Submitted by the Author:	24-May-2022
Complete List of Authors:	Crawford, Mike; Imperial College London, Psychological Medicine<br>Leeson, Verity; Imperial College London<br>McQuaid, Aisling; Imperial College London<br>Samuel, Oluwaseun; Imperial College London<br>King, Jacob D; Imperial College London<br>Di Simplicio, Martina; Imperial College London<br>Tyrer, Peter; Imperial College London, Psychological medicine<br>Tyrer, Helen; Imperial College London<br>Watt, Richard; University College London, Epidemiology and Public Health<br>Barnicot, Kirsten; City University of London
**Primary Subject Heading**:	Mental health
Secondary Subject Heading:	Mental health
Keywords:	COVID-19, Anxiety disorders < PSYCHIATRY, Clinical trials < THERAPEUTICS

Severe COVID anxiety among adults in the United Kingdom: protocol for a cohort study and nested feasibility trial of modified Cognitive Behaviour Therapy for Health Anxiety.

Mike J Crawford, Verity C Leeson, Aisling McQuaid, Oluwaseun Samuel, Jacob D King, Martina Di Simplicio, Peter Tyrer, Helen Tyrer, Richard G Watt, Kirsten Barnicot.

ABSTRACT

Introduction

Some people are so anxious about COVID that it impairs their functioning. However, little is known about the course of severe COVID anxiety or what can be done to help people who experience it.

Methods and analysis

Cohort study with a nested feasibility trial with follow-up at three and six months. We recruited 306 people who were aged 18 and over, lived in the United Kingdom and had severe COVID anxiety (indicated by a score of nine or more on the Coronavirus Anxiety Scale). To take part in the nested feasibility trial, participants also had to have a score of 20 or more on the Short Health Anxiety Inventory. We excluded people from the trial if they had had COVID-19 within the previous four weeks, if they were currently self-isolating or if they were already receiving psychological treatment.

We publicised the study nationally through adverts, social media and posts on chat boards. We also recruited participants via clinicians working in primary and secondary care NHS services in London. All those in the active arm will be offered five to ten sessions of remotely delivered modified Cognitive Behaviour Therapy for Health Anxiety (CBT-HA). We will examine the proportion of participants who remain above threshold on the Coronavirus Anxiety Scale at three and six months and factors that influence levels of COVID anxiety over six months using mixed-effects logistic regression. The key feasibility metrics for the nested trial are the level of uptake of CBT-HA and the rate of follow-up.

Ethics and dissemination

Approved by Leicester Central Research Ethics Committee (reference: 20/EM/0238). The results of the study will be published in peer-reviewed scientific journals.

For peer review only - http://bmjopen.bmj.com/site/about/guidelines.xhtml

For peer review only

Trial registration: International Standard Randomised Control Trial Number Register - ISRCTN14973494

Strengths of the study

- The first study to explore the feasibility of using a modified form of CBT-HA for people with severe COVID anxiety.
- Use of remote methods for data collection and delivering the intervention, which enable participation by those who are unable to attend face-to-face meetings.

Limitations of the study

- The study design means that we will not be able to estimate the prevalence of severe COVID anxiety in the general population.
- Reliance on remote methods for recruitment and follow-up may impact on the follow up rate.

INTRODUCTION

The COVID-19 pandemic is having a major impact on mental health throughout the world.(1, 2) In the UK, there was a marked increase in the proportion of people experiencing anxiety after the first wave of the pandemic,(3-5) a trend which has also been seen in many other countries.(6) Evidence from longitudinal studies in the UK suggest that a range of factors including social isolation, job insecurity and financial problems were important.(3, 7) Qualitative data collected during his period reinforce the importance of these factors and have also highlighted disruption to work and other routines and a sense of lack of control.(8, 9)

Fear is an appropriate response to a new infectious disease like COVID-19 that threatens health, lives and livelihoods. At the start of the pandemic it was unclear how many people

For peer review only - http://bmjopen.bmj.com/site/about/guidelines.xhtml

were likely to become infected, what the case fatality rate was and to what extent people who survived the initial illness would go on to experience long-term negative effects. Under these circumstances, fearfulness and anxiety are to be expected. Indeed, research conducted in the early phase of the pandemic found that fear of COVID-19 can be adaptive, resulting in greater adherence to public health measures aimed at limiting the spread of the virus.(10, 11) However, as the pandemic has progressed it has become clear that some people have become so anxious about COVID that it is impacting on their mental health and social functioning. People with severe COVID anxiety are more likely to report harmful use of alcohol and drugs, hopelessness and suicidal thoughts.(12, 13) To date, researchers investigating this topic have used slightly different definitions of 'COVID anxiety'. One of the first teams to investigate the subject, led by Lee and colleagues in Virginia (USA), used a narrow definition based on the cognitive, behavioural and physiological components of anxiety.(12-13) More recently teams such as Spada and colleagues in London (UK), have adopted a broader definition that incorporates a wider range of behavioural responses including avoidance, checking and threat monitoring.(14, 15) Nonetheless, all those investigating COVID anxiety agree that core symptoms are those found in other anxiety disorders, and it impacts on behaviour and social functioning.

At the start of the pandemic it was unclear what could be done to help people who appeared to have severe COVID anxiety. In the absence of evidence, governments, professional organisations and mental health services recommended general public mental health measures, such as maintaining contact with family and friends, keeping regular routines and sleeping times, and avoiding drug use and excessive use of alcohol.(16, 17). However, in our discussions with patients referred to mental health services who had severe COVID anxiety, we found that some appeared to be suffering from health anxiety. Health anxiety, formerly referred to as hypochondriasis, is a condition in which people become overwhelmed by fears of becoming ill.(18)

Health anxiety was a major contributor to poor mental health during previous pandemics.(19, 20) Emerging evidence from public surveys in Europe, Asia and North America have shown it is also contributing to poor mental health during the COVID-19 pandemic.(21-23) People with health anxiety may repeatedly search the internet and other

For peer review only - http://bmjopen.bmj.com/site/about/guidelines.xhtml

media for information about health conditions, leading to mental distress and impaired functioning.(24, 25) People with health anxiety may misinterpret bodily sensations as symptoms of a physical illness and seek reassurance from others.(26) However, symptom monitoring and reassurance seeking may increase the amount of time that people think about being unwell and increase their level of anxiety.(18) During the COVID pandemic people may be at greater risk of health anxiety because of widespread media coverage, misinformation about COVID and uncertainty about the course of the pandemic.(27) In addition to this people are being actively encouraged to stay alert for signs of infection and get tested for COVID-19 as soon as possible if even mild signs of infection occur. This approach is an important public health strategy for limiting the transmission of COVID,(28) but it may also increase the likelihood of people who are predisposed to being anxious about their health becoming severely anxious to the extent that reduces their quality of life and impacts on their ability to function.(20)

The link between health anxiety and poor mental health seen in previous pandemics, is important because health anxiety is treatable. Cognitive Behavioural Therapy for Health Anxiety (CBT-HA) leads to long-term reductions in levels of health anxiety, generalised anxiety and depression.(29, 30) In recent years the intervention has been successfully modified to enable it to be delivered remotely.(31)

As social distancing measures are relaxed and people are able to resume social and occupational roles, it is essential to understand the course and consequences of severe COVID anxiety and examine interventions that could improve people's mental health and functioning. However, at this stage, we do not know the course of COVID anxiety or the contribution that health anxiety plays in maintaining it. While CBT-HA is effective under normal circumstances, we need to find out if it is an appropriate intervention to offer during a global pandemic, when people continue to be asked to be vigilant in spotting the signs of infection in an effort to prevent transmission of the virus.

Objectives

This study aims to reduce current gaps in knowledge and contribute to the development of a more effective response to health anxiety during pandemics. The study objectives are to;

For peer review only - http://bmjopen.bmj.com/site/about/guidelines.xhtml

1) Assess the impact of severe COVID anxiety on people's social functioning and health-related quality of life,

2) Examine the course of severe COVID anxiety over a six-month period,

3) Identify factors that influence the course of severe COVID anxiety, and

4) Test the feasibility of a randomised controlled trial of CBT-HA for people with severe COVID anxiety and health anxiety aimed at improving mental health and social functioning.

METHODS AND ANALYSIS

The COVID Anxiety Project is a cohort study with a nested feasibility trial. People taking part in the study will be followed up for six months. The trial is a researcher-blind, parallel-arm, randomised controlled feasibility trial, which is compliant with Standard Protocol Items: Recommendations for Interventional Trials guidelines.(32)

Study population and sample

Study participants were recruited from the general public in the United Kingdom and from those in contact with primary care and secondary care mental health services in London. We aimed to recruit people who self-identified as being anxious about COVID-19 using a broad range of methods including social media sites (Facebook, Reddit, Instagram and Twitter) and mental health charities (MQ research and Anxiety UK). We publicised the study via 19 primary care practices in north and west London and through colleagues working in secondary care mental health services in Central and North West London NHS Foundation Trust, City and East London NHS Foundation Trust and West London NHS Trust.

To be eligible to take part in the cohort study, potential participants had to be aged 18 or over, live in the United Kingdom and score nine or more on the Coronavirus Anxiety Scale.(12) We used a threshold of nine or more on the CAS because this identifies those with moderate or severe functional impairment with 90% sensitivity, 85% specificity, and a false positive rate of 15%.(12)

We excluded people who had a current or previous diagnosis of a psychotic mental disorder. To take part in the nested feasibility trial, participants also had to have a score of 20 or more on the Short Health Anxiety Inventory (SHAI).(33) We excluded people from the feasibility trial if, at the time of the baseline assessment, they:

- had had COVID-19 in the prior four weeks (defined as a positive antigen test or a diagnosis by a clinician),

For peer review only - http://bmjopen.bmj.com/site/about/guidelines.xhtml

- were self-isolating on the advice of a doctor or the NHS Test and Trace service; or
- were already receiving psychological treatment for any condition.

The cohort study will be made up of all study participants with the exception of those offered CBT-HA as part of the feasibility trial.

Recruitment and follow-up

Participant flow through the study is presented in figure 1. We posted adverts on social media sites and websites of mental health charities and asked staff working in primary and secondary care NHS services to direct potential participants to a dedicated study website that was hosted by Qualtrics (www.qualtrics.com). The site included a copy of the Participant Information Sheet. Potential participants were asked to sign and date an online consent form, confirm their eligibility to take part in the study, and complete the Coronavirus Anxiety Scale. The survey was designed so that it could only be completed once from any one IP address.(34) Those who scored nine or more on the scale and met other eligibility criteria were invited to complete the baseline survey. Those who were not eligible for the study were directed to a webpage which listed sources of mental health support.

We identified potential participants for the feasibility trial from their responses to the baseline survey. We aimed to recruit participants according to the capacity of study therapists to deliver CBT-HA to those in the active arm of the trial. When therapists had capacity to work with more people, we emailed those who recently joined the cohort study and met the additional eligibility criteria a copy of an additional Participant Information Sheet and directed them to an online consent form. A researcher contacted these participants by telephone to answer any queries they had about the study, confirm that they met study eligibility criteria, support them to complete the online consent form and counter sign it. The researcher then contacted the trial manager who randomised the participant. Any participant who was approached to take part in the randomised trial and was ineligible or declined to take part, remained in the cohort study and will be asked to complete subsequent follow-up surveys.

Following attempts by what appeared to be automated survey-takers or ‘bots’ to take part in the study, we added a CAPTCHA question and a ‘trap’ question.(34, 35) The ‘trap’ question provided a range of valid and non-valid but plausible options for how the potential participant had heard about the study, with only those providing a valid response being invited to complete the baseline survey.

For peer review only - http://bmjopen.bmj.com/site/about/guidelines.xhtml

Between February 2021 and September 2021, we recruited 306 participants to the study. Of these, 127 (41.5%) met inclusion criteria for the feasibility trial. Among 108 participants offered a place on the trial 40 (37.0%) accepted and were randomised. The total sample for the cohort study is 285 comprising 179 who were ineligible for the trial, 19 who were eligible but not offered a place due to limited capacity of therapists to take on new patients, 68 who were offered a place on the trial but declined it and 19 people who were randomised to treatment as usual.

All participants in the cohort study and the feasibility trial will be sent an automated email to ask them to complete the three- and six-month follow-up survey. Those who have not responded after one week will be sent reminders. Trial participants who do not complete follow-up interviews will also be contacted by researchers by email (or telephone if contact number is available) with requests to complete the follow-up surveys.

Randomisation and blinding

We generated a randomisation list using the independent web-based service 'sealed envelope' (https://www.sealedenvelope.com/simple-randomiser/v1/lists). We stratified according to score on the short Health Anxiety Inventory (≤24 and ≥25) and the Dependent Personality Questionnaire (≤11 and ≥12) using a ratio of CBT-HA to control treatment of 1:1. We included the Dependent Personality Questionnaire as a stratification variable because previous research has demonstrated that the presence of dependent personality traits may influence the uptake and impact of CBT-HA.(36)

Throughout the study, the randomisation list will be encrypted and stored electronically by the trial manager. At the end of the study the randomisation list will be unencrypted and placed in the Trial Master File. The trial manager generated the allocation of each new trial participant by allocating them to the next treatment arm in the predetermined list. The participant was informed of their allocation by telephone or email, and the first therapy session scheduled for those allocated to CBT-HA. Researchers will have very little contact with participants other than to encourage them to complete follow up surveys. In the unlikely event that researchers speak to a study participant after they have been randomised, they will start by reminding them of the need to make sure they are not aware of whether the participant was offered CBT-HA or control treatment.

Assessments

The timing and sequence of all assessments are summarised in table 1.

For peer review only - http://bmjopen.bmj.com/site/about/guidelines.xhtml

Screening assessment

To take part in the cohort study, potential participants had to score nine or more on the Coronavirus Anxiety Scale (CAS).(12, 13) Developed by Sherman Lee in the USA, the scale was designed to provide a brief and reliable measure of the level of COVID-related anxiety that people experience. It comprises five questions on the frequency of anxious thoughts, somatic symptoms and sleep disturbance triggered by reading, thinking or hearing about COVID.

Baseline assessment and covariates

At baseline we collected self-reported data on demographic factors (age, gender, ethnicity, household composition, occupational status), physical health (current medical conditions), and exposure to COVID (whether they had had COVID, whether they had been admitted to hospital with COVID). We asked participants whether they live with or care for someone that might get seriously ill if infected with COVID because of their health or age and whether someone in their family or a close friend had been in hospital with COVID.

We also asked participants questions about behaviours intended to reduce the risk of exposure to COVID. These questions were developed with the help of the members of the Lived Experience Advisory Panel and covered five behaviours which people had used or heard of others using in an effort to avoid catching COVID: staying at home, avoiding shops, washing or discarding letters and parcels, increased handwashing and increased washing of clothes. People who lived with school age children were also asked whether they had stopped them attending school because of concerns about COVID.

We assessed self-reported functioning using the Work and Social Adjustment scale (WSAS)(37) and health-related quality of life using the EQ-5D-3L.(38) We assessed mental health using the Patient Health Questionnaire-9,(39) Generalised Anxiety Disorder 7-item scale,(40) Obsessive -Compulsive Inventory -Revised,(41) the short form of the Health Anxiety Inventory (SHAI),(33) the Standardised Assessment of Personality – Abbreviated Scale,(42) the Dependent Personality Questionnaire,(43) use of alcohol (AUDIT-C)(44) and a single-question screening test for drug use.(45) Finally, we asked participants to indicate admissions to hospital, contacts with primary and secondary care services using items from the Adult Service User Schedule.(46)

Follow-up surveys

For peer review only - http://bmjopen.bmj.com/site/about/guidelines.xhtml

All those taking part in the cohort study or the feasibility trial are asked to complete follow-up surveys three and six months after the baseline survey. The content of these surveys was similar to the baseline survey, except that we did not repeat the personality assessments (SAPAS and DPQ), and we added a question at six months on whether people had received a COVID-19 vaccination (see table 1).

In recognition of their time and support, all those who completed a baseline interview were sent a £10 gift voucher and a further £20 voucher will be offered to those that complete the six-month follow-up interview.

Serious adverse events

Adverse events that are identified in the survey (e.g. worsening or newly emerging clinical phenomena) will be added to an adverse event log. Data on Serious Adverse Event (SAE) including death, hospitalisation and life-threatening events are recorded at baseline, and three and six month follow-up. Therapists will also be regularly reminded that they should report all serious adverse events to the clinical trial team. SAEs will be recorded on a non-CTIMP SAE form and forwarded within 24 hours to the Chief Investigator and sponsor if judged to be unexpected and related to the research procedures. The Research Ethics Committee will be notified within 15 days of the Chief Investigator becoming aware of any such SAE.

Interventions

All those who agree to take part in the feasibility trial will be sent a self-help booklet developed by staff at Central and North West London NHS Foundation Trust that is derived from the work of Russ Harris on Acceptance and Commitment Therapy,(47) and aims to help people cope with the COVID crisis by building resilience, identifying and implementing healthy coping strategies and general advice on health and wellbeing.(48) All participants will be able access treatment as usual, which includes access to NHS primary care services and referral on to specialist services if required.

All those in the active arm of the feasibility trial will be offered five to ten sessions of modified Cognitive Behavioural Therapy for Health Anxiety (CBT-HA) based on a published treatment manual.(49) Therapists will start by taking a detailed history of the person's thoughts and fears about COVID and exploring their beliefs about the impact of COVID and their ability to cope, in order to develop a formulation. Therapists will seek to identify behaviours such as symptom monitoring and reassurance seeking that could be maintaining

For peer review only - http://bmjopen.bmj.com/site/about/guidelines.xhtml

the person's anxiety. They will then use Socratic dialogue, diary keeping and behavioural experiments to help participants recognise the links between their thoughts and behaviour and explore ways to reduce their anxiety.

All CBT-HA sessions will be delivered by phone or videoconferencing, in accordance with the participant's preference. Sessions will generally last between 30 and 50 minutes and be offered on a weekly or fortnightly basis. Therapists will consider delaying their final session in order to give people an opportunity to use the techniques and skills they have learned and reinforce the changes they have made. Sessions will be delivered in keeping with a published treatment manual but modified to meet the difficulties that people in the study are experiencing. Sessions will be supplemented by booklets summarising the causes of health anxiety and how patients can begin to overcome their fears.

Each therapist will record the number and length of sessions they offer participants so that we can calculate the proportion of people who are offered CBT-HA who start and complete treatment and the number of sessions that people receive.

All therapists have a degree in a health-related subject and have previous experience of delivering psychological treatments. All therapists attended a 90-minute training session by Dr Helen Tyrer and will receive fortnightly supervision sessions delivered by Dr Tyrer.

The nature of modifications to the original CBT-HA model will be recorded during the study and presented alongside the results of the feasibility trial.

Sample size

We aimed to recruit sufficient numbers of participants to the cohort study in order to randomise 40 participants to the feasibility trial, which is a typical size for such a trial.(50) A sample of 40 participants would enable us to detect study consent and intervention completion rates of 50%, with 95% confidence intervals of +/-11% and +/- 15% respectively. Assuming a rate of follow-up at six months of 75%, our cohort of 285 study participants will generate valid data on 213 people. A sample of 196 participants in the cohort study will enable us to estimate a remission rate of 15% +/- 5%, with 95% confidence.(51)

Data analysis

We will start by examining the characteristics of the study sample, including the prevalence of coexisting physical and mental health conditions, levels of disturbance in work and social functioning, quality of life, and actions taken to try to avoid catching COVID using simple descriptive statistics. For the cohort study, we will examine the proportion who remain

For peer review only - http://bmjopen.bmj.com/site/about/guidelines.xhtml

above threshold for severe COVID anxiety (9 or above on the CAS) and for COVID anxiety (5 or more on the CAS) at three and six months after completion of the baseline survey. We will examine factors that influence changes in CAS scores over this period using linear regression analysis. The analysis will be performed in two stages. Initially the separate association between each factor and CAS at each outcome timepoint will be assessed separately. CAS score at baseline will be included in all these analyses, as the inclusion of this factor will mean that the analyses reflect factors associated with change in CAS from baseline. The second stage of the analysis will examine the joint association between the factors and CAS score in a multivariable analysis. To restrict the number of variables in this stage of the analysis, only factors showing some association with the outcome ($p<0.2$) from the first stage of the analysis will be considered in the multivariable analyses. A selection procedure (e.g. backwards selection) will be considered to identify factors significantly associated with the outcome and to be included in the final model.

We anticipate that there will be little missing data because the platform we designed for data entry requires participants to complete every item of a questionnaire before moving on to the next section. However where the number of missing items on a given measure is 20% or less, then the missing value for the items will be substituted by the individual's mean score for the remaining items on the scale. If there are more than 20% missing items in the scale the outcome measure will not be calculated for the participant at that time point.

Our criteria for determining the success of the feasibility study, which are based on thresholds used in other recent feasibility and pilot studies,(52, 53) are: recruitment of at least 32 participants (80% of the target study sample of 40 participants), uptake of the intervention by at least 60% of participants in the active arm of the trial, and completion of follow-up interviews at six months by 75% of study participants. All data will be analysed in SPSS version 2.0 (SPSS Inc., Chicago IL) and Stata version 16.1.(54)

Patient and public involvement

Patients and the public contributed to the development of this proposal. Charlotte Green, who chaired the Patient & Carers Research Interest group at CNWL NHS Foundation Trust, helped develop the proposal and write the lay summary. We presented plans for the study to members of a local patient research interest group via Zoom. Members expressed strong support for the study and eight agreed to join a Lived Experience Advisory Group. Feedback

For peer review only - http://bmjopen.bmj.com/site/about/guidelines.xhtml

from the group led to us to adding new questions on the impact of COVID anxiety. Members made suggestions for how to access potential participants, which we adopted. Members of the group also highlighted the importance of tailoring CBT-HA to the needs of individual patients, including making time for them to discuss broader concerns about their mental health and the impact of the pandemic. Members of the Lived Experience Advisory Group will help us prepare a plain English summary of study findings, which we will make available to all study participants and other members of the public through the study website.

ETHICS AND DISSEMINATION

Plans for the study were approved by Leicester Central Research Ethics Committee (reference: 20/EM/0238). The study will be conducted in accordance with the recommendations for physicians involved in research on human subjects adopted by the 18th World Medical Assembly, Helsinki 1964 and later revisions.

All research activity will be carried out after participants have given consent, and this documented on either an electronic or paper form. For the cohort study, the consent forms will be completed independently by the participant without researcher support but a member of the study team will be available to answer any questions prior to consent being given. Individual consent completed for the cohort study will be linked in Qualtrics to the questionnaires completed by the same participant at each timepoint in the cohort study to ensure that the person that gave consent is completing the assessments.

Separate consent to take part in the clinical trial will be completed by the participant during a telephone call with a member of the research team. The member of the research team that facilitates the completion of the consent process on the telephone will countersign the consent form to document that they were present.

Completed electronic consent forms will be downloaded from Qualtrics, printed and stored in a Master File. A copy of their completed consent form(s) will be provided to each participant. Any original paper consent forms will also be stored in the Master File. All participants are free to withdraw at any time from the cohort study or clinical trial without giving reasons and without prejudicing further treatment.

The Chief Investigator will preserve the confidentiality of participants taking part in the study and is registered under the Data Protection Act. Data from the consent forms and

For peer review only - http://bmjopen.bmj.com/site/about/guidelines.xhtml

questionnaires, including identifiable personal data, will be stored on Qualtrics, an online survey tool. Qualtrics has ISO 27001 certification, meaning that it meets the international standard for managing confidential and sensitive data. All data transfers to Imperial College London use TLS encryption. Imperial College will be the data controller and there is a service-level agreement in place between Imperial College and Qualtrics.

Where questionnaires are administered during a telephone interview rather than online, the researcher will type the responses into Qualtrics. Other personal data that is recorded during the study (e.g. therapy notes from CBT sessions) will be typed directly onto the secure server of Imperial College London and will be identified using only the participant identifier. Qualtrics allows data erasure and all study data will be deleted from Qualtrics at the end of the study, following extraction for analysis.

Paper documentation will be minimal during this research. An option to complete paper consent form and screening questionnaires was offered but not taken up by any participant.

The results of the study will be published in peer-reviewed scientific journals. We anticipate three key papers examining baseline data, follow-up data and the results of the feasibility trial. We will work with members of our Lived Experienced Advisory Panel to prepare a lay summary of findings from the study which we will post on the study website and will email all study participants the link to this summary.

DISCUSSION

This study aims to build on the results of previous research that has highlighted the impact of COVID anxiety on mental health during the current pandemic. By collecting prospective data on a sample of people who have high levels of COVID anxiety, we will examine its course over a six-month period. Having collected detailed information on demographic and clinical factors at baseline we will be able to examine the role these factors play in maintaining COVID anxiety over time. By conducting a nested feasibility trial we will be able to examine the uptake and acceptability of an intervention for health anxiety, a condition which is strongly linked to anxiety during pandemics and has the potential to benefit people who experience these problems.

This study has a number of strengths and limitations. We believe that it is the first to examine the feasibility of offering people with severe COVID anxiety a psychological

For peer review only - http://bmjopen.bmj.com/site/about/guidelines.xhtml

intervention for their distress. While many studies have examined the prevalence and aetiology of mental health problems during the COVID crisis, far fewer have started to explore the impact of interventions for mental health problems, and we are not aware of any studies examining the impact of CBT-HA for those who have coexisting health anxiety. By using remote methods for collecting study data and delivering the intervention, we have been able to include people in the study who were so anxious about COVID that they were unlikely to attend face-to-face appointments.

Because we did not use a general population sample for the survey, we will not be able to estimate the prevalence of severe COVID anxiety in the general population. Our reliance on remote means for data collection may have put off some people from taking part in the study. We did make provision for people taking part in the study via telephone interviews, telephone-based therapy and signing paper copies of consent forms. However, people with limited or no access to the internet may have been put off from taking part.

We are using the Coronavirus Anxiety Scale as our primary outcome measure.(12) Since the start of the study, other measures have been developed which assess a wider range of behaviours that are associated with COVID anxiety.(14) While we are also asking study participants to describe the frequency of avoidant behaviours associated with COVID anxiety, the psychometric properties of these questions have not be tested.

Our reliance on remote methods for recruitment and following up study participants may have limited our ability to develop a rapport with them and this could, in turn, affect our response rate.(55) We will keep in touch with study participants by email and offer people an honoraria to complete the follow-up surveys in an effort to minimise loss to follow up. While the nested clinical trial is large enough to examine the feasibility of a future explanatory trial of CBT-HA for people with severe COVID anxiety it has not been powered to examine the clinical effectiveness of this intervention.

Figure 1. Study flow diagram

For peer review only

For peer review only - http://bmjopen.bmj.com/site/about/guidelines.xhtml

AUTHOR AFFILIATIONS

1) Professor Mike J Crawford (Corresponding author). Division of Psychiatry, Imperial College London, Commonwealth Building, Hammersmith Hospital Campus, Du Cane Road, London W12 0NN, UK. m.crawford@imperial.ac.uk
2) Dr Verity Leeson. Division of Psychiatry, Imperial College London, Commonwealth Building, Hammersmith Hospital Campus, Du Cane Road, London W12 0NN, UK. v.leeson@imperial.ac.uk
3) Ms Aisling McQuaid. Division of Psychiatry, Imperial College London, Commonwealth Building, Hammersmith Hospital Campus, Du Cane Road, London W12 0NN, UK. a.mc-quaid@imperial.ac.uk
4) Ms Oluwaseun Samuel. Division of Psychiatry, Imperial College London, Commonwealth Building, Hammersmith Hospital Campus, Du Cane Road, London W12 0NN, UK. o.samuel@imperial.ac.uk
5) Dr Jacob King. Division of Psychiatry, Imperial College London, Commonwealth Building, Hammersmith Hospital Campus, Du Cane Road, London W12 0NN, UK. j.king20@imperial.ac.uk
6) Dr Martina Di Simplicio. Division of Psychiatry, Imperial College London, Commonwealth Building, Hammersmith Hospital Campus, Du Cane Road, London W12 0NN, UK. m.di-simplicio@imperial.ac.uk
7) Professor Peter Tyrer. Division of Psychiatry, Imperial College London, Commonwealth Building, Hammersmith Hospital Campus, Du Cane Road, London W12 0NN, UK. p.tyrer@imperial.ac.uk
8) Dr Helen Tyrer, Division of Psychiatry, Imperial College London, Commonwealth Building, Hammersmith Hospital Campus, Du Cane Road, London W12 0NN, UK. htyrer07@gmail.com
9) Professor Richard G Watt Department of Epidemiology & Public Health, University College London, 1-19 Torrington Place, London, WC1E 7HB, UK. r.watt@ucl.ac.uk
10) Paul Bassett, Statsconsultancy Ltd, Amersham, Buckinghamshire, UK. paul@statsconsultancy.co.uk
11) Dr Kirsten Barnicot. Division of Health Services Research and Management, City, University of London, Northampton Square, London EC1V 0HB, UK. Kirsten.Barnicot@city.ac.uk

Authors' contributions

MC is the Chief Investigator who conceived the study and led the design of the study and the study protocol and preparation of this manuscript. VL helped design the study protocol, obtained study approvals and contributed to the preparation of this manuscript. AMcQ, OS and JK recruited study participants contributed to the design of the study and the study

For peer review only - http://bmjopen.bmj.com/site/about/guidelines.xhtml

protocol and preparation of this manuscript. HT helped to develop the study intervention and contributed to the preparation of this manuscript. MdS, RW, PT and KB, contributed to the design of the study, the development of the study protocol and the preparation of this manuscript. JK and MC developed the statistical analysis plan. All authors read and approved the final manuscript.

Acknowledgements

We are grateful to the members of a Combined Independent Oversight Committee which will oversee project governance, data management and review safety data (Dr John Green (chair), Professor Khalida Ismail and Mr Robert Koch). We thank Paul Bassett, freelance statistican, for reviewing the protocol and revising a draft of the Statictical Analysis Plan. We also thank members of the lived experience reference group including Manisha Ahya, Anjie Chhapia, Charlotte Green, Sandra Jayacodi, Niruben Patel and Vikas Sharma.

Funding statement

This research is supported by funding from the NIHR Imperial Biomedical Research Centre, the National Institute for Health Research and an NIHR Senior Investigator Application held by Professor Crawford (NF-SI-0515-10006). The views expressed are those of the authors and not necessarily those of the NIHR or the Department of Health and Social Care.The sponsor of the trial is Imperial College London.

COMPETING INTERESTS STATEMENT

Dr Helen Tyrer is author of a book on Cognitive Behaviour Therapy for treating people with Health Anxiety. Other authors declare that they have no competing interests.

For peer review only

For peer review only - http://bmjopen.bmj.com/site/about/guidelines.xhtml

REFERENCES

1. World Health Organization. Mental Health Considerations During COVID-19 Outbreak Geneva: World Health Organization, 2020.

2. Thombs BD, Bonardi O, Rice DB, Boruff JT, Azar M, He C, et al. Curating evidence on mental health during COVID-19: A living systematic review. Journal of Psychosomatic Research. 2020;133:110113.

3. Pierce M, Hope H, Ford T, Hatch S, Hotopf M, John A, et al. Mental health before and during the COVID-19 pandemic: a longitudinal probability sample survey of the UK population. The Lancet Psychiatry. 2020;7:883-92.

4. Jia R, Ayling K, Chalder T, Massey A, Broadbent E, Coupland C, et al. Mental health in the UK during the COVID-19 pandemic: cross-sectional analyses from a community cohort study. BMJ Open. 2020;10:e040620.

5. Santabarbara J, Lasheras I, Lipnicki DM, Bueno-Notivol J, Perez-Moreno M, Lopez-Anton R, et al. Prevalence of anxiety in the COVID-19 pandemic: An updated meta-analysis of community-based studies. Progress in Neuro-Psychopharmacology & Biological Psychiatry. 2021;109:110207.

6. Santomauro DF, Herrera AM, Shadid J, Zheng P, Ashbaugh C, Pigott DM, Abbafati C, Adolph C, Amlag JO, Aravkin AY, Bang-Jensen BL. Global prevalence and burden of depressive and anxiety disorders in 204 countries and territories in 2020 due to the COVID-19 pandemic. The Lancet. 2021;398:1700-12.

7. McPherson KE, McAloney-Kocaman K, McGlinchey E, Faeth P, Armour C. Longitudinal analysis of the UK COVID-19 Psychological Wellbeing Study: Trajectories of anxiety, depression and COVID-19-related stress symptomology. Psychiatry Research. 2021;304:114138.

8. Burton A, McKinlay A, Aughterson H, Fancourt D. Impact of the COVID-19 pandemic on the mental health and well-being of adults with mental health conditions in the UK: a qualitative interview study. Journal of Mental Health. 2021:1-8.

For peer review only - http://bmjopen.bmj.com/site/about/guidelines.xhtml

9. McKinlay AR, May T, Dawes J, Fancourt D, Burton A. 'You're just there, alone in your room with your thoughts': a qualitative study about the psychosocial impact of the COVID-19 pandemic among young people living in the UK. BMJ Open. 2022;12:e053676.

10. Harper CA, Satchell LP, Fido D, Latzman RD. Functional Fear Predicts Public Health Compliance in the COVID-19 Pandemic. International Journal of Mental Health and Addiction. 2021: 19:1875-88.

11. Chen SX, Ng JCK, Hui BPH, Au AKY, Wu WCH, Lam BCP, et al. Dual impacts of coronavirus anxiety on mental health in 35 societies. Scientific Reports. 2021;26:1-1.

12. Lee SA. Coronavirus Anxiety Scale: A brief mental health screener for COVID-19 related anxiety. Death Studies. 2020;44:393-401.

13. Lee SA, Mathis AA, Jobe MC, Pappalardo EA. Clinically significant fear and anxiety of COVID-19: A psychometric examination of the Coronavirus Anxiety Scale. Psychiatry Research. 2020;290:113112.

14. Nikcevic AV, Spada MM. The COVID-19 anxiety syndrome scale: Development and psychometric properties. Psychiatry Research. 2020;292:113322.

15. Albery IP, Spada MM, Nikcevic AV. The COVID-19 anxiety syndrome and selective attentional bias towards COVID-19-related stimuli in UK residents during the 2020-2021 pandemic. Clin Psychol Psychother. 2021;28:1367-78.

16. Department of Health and Social Care. New advice to support mental health during coronavirus outbreak London: Department of Health and Social Care; 2020 Available from: https://www.gov.uk/government/news/new-advice-to-support-mental-health-during-coronavirus-outbreak. Accessed 18/05/2022.

17. World Health Organization. Looking after our mental health Geneva: WHO; 2020. Available from: https://www.who.int/campaigns/connecting-the-world-to-combat-coronavirus/healthyathome/healthyathome---mental-health. Accessed 18/05/2022.

18. Tyrer P, Eilenberg T, Fink P, Hedman E, Tyrer H. Health anxiety: the silent, disabling epidemic. BMJ. 2016;353:i2250.

For peer review only - http://bmjopen.bmj.com/site/about/guidelines.xhtml

19. Wheaton MG, Abramowitz JS, Berman NC, Fabricant LE, Olatunji BO. Psychological predictors of anxiety in response to the H1N1 (swine flu) pandemic. doi:10.1007/s10608-011-9353-3. Cognitive Therapy and Research. 2012;36:210-8.

20. Asmundson GJG, Taylor S. How health anxiety influences responses to viral outbreaks like COVID-19: What all decision-makers, health authorities, and health care professionals need to know. Journal of Anxiety Disorders. 2020;71:102211.

21. Jungmann SM, Witthoft M. Health anxiety, cyberchondria, and coping in the current COVID-19 pandemic: Which factors are related to coronavirus anxiety? Journal of Anxiety Disorders. 2020;73:102239.

22. Nikcevic AV, Marino C, Kolubinski DC, Leach D, Spada MM. Modelling the contribution of the Big Five personality traits, health anxiety, and COVID-19 psychological distress to generalised anxiety and depressive symptoms during the COVID-19 pandemic. Journal of Affective Disorders. 2021;279:578-84.

23. Ozdin S, Bayrak Ozdin S. Levels and predictors of anxiety, depression and health anxiety during COVID-19 pandemic in Turkish society: The importance of gender. International Journal of Social Psychiatry. 2020;66:504-11.

24. Te Poel F, Baumgartner SE, Hartmann T, Tanis M. The curious case of cyberchondria: A longitudinal study on the reciprocal relationship between health anxiety and online health information seeking. Journal of Anxiety Disorders. 2016;43:32-40.

25. McMullan RD, Berle D, Arnaez S, Starcevic V. The relationships between health anxiety, online health information seeking, and cyberchondria: Systematic review and meta-analysis. Journal of Affective Disorders. 2019;245:270-8.

26. Olatunji BO, Deacon BJ, Abramowitz JS, Valentiner DP. Body vigilance in nonclinical and anxiety disorder samples: Structure, correlates, and prediction of health concerns. Behavior Therapy. 2007;38:392-401.

27. Tyrer P. COVID-19 health anxiety. World Psychiatry. 2020;19:307-8.

28. Girum T, Lentiro K, Geremew M, Migora B, Shewamare S. Global strategies and effectiveness for COVID-19 prevention through contact tracing, screening, quarantine, and isolation: a systematic review. Tropical Medicine and Health. 2020;48:1-5.

For peer review only - http://bmjopen.bmj.com/site/about/guidelines.xhtml

29. Tyrer P, Cooper S, Salkovskis P, Tyrer H, Crawford M, Byford S, et al. Clinical and cost-effectiveness of cognitive behaviour therapy for health anxiety in medical patients: a multicentre randomised controlled trial. The Lancet. 2014;383:219-25.

30. Cooper K, Gregory JD, Walker I, Lambe S, Salkovskis PM. Cognitive Behaviour Therapy for Health Anxiety: A Systematic Review and Meta-Analysis. Behavioural and Cognitive Psychotherapy. 2017;45:110-23.

31. Morriss R, Patel S, Malins S, Guo B, Higton F, James M, et al. Clinical and economic outcomes of remotely delivered cognitive behaviour therapy versus treatment as usual for repeat unscheduled care users with severe health anxiety: a multicentre randomised controlled trial. BMC Medicine. 2019;17:16.

32. Chan AW, Tetzlaff JM, Altman DG, Laupacis A, Gotzsche PC, Krleza-Jeric K, et al. SPIRIT 2013 statement: defining standard protocol items for clinical trials. Annuls of Internal Medicine. 2013;158:200-7.

33. Salkovskis PM, Rimes KA, Warwick HM, Clark DM. The Health Anxiety Inventory: development and validation of scales for the measurement of health anxiety and hypochondriasis. Psychological Medicine. 2002;32:843-53.

34. Storozuk A, Ashley M, Delage V, Maloney E. Got Bots? Practical Recommendations to Protect Online Survey Data from Bot Attacks. The Quantitative Methods for Psychology. 2020;16:472–81.

35. Liu M, Wronski L. Trap questions in online surveys: Results from three web survey experiments. International Journal of Market Research. 2018;60:32-49.

36. Sanatinia R, Wang D, Tyrer P, Tyrer H, Crawford M, Cooper S, et al. Impact of personality status on the outcomes and cost of cognitive-behavioural therapy for health anxiety. The British Journal of Psychiatry : the journal of mental science. 2016;209:244-50.

37. Mundt JC, Marks IM, Shear MK, Greist JH. The Work and Social Adjustment Scale: a simple measure of impairment in functioning. The British Journal of Psychiatry. 2002;180:461-4.

38. Brooks RG. EuroQoL- the current state of play. Health Policy. 1996;37:53-72.

For peer review only - http://bmjopen.bmj.com/site/about/guidelines.xhtml

39. Kroenke K, Spitzer RL, Williams JB. The PHQ-9: validity of a brief depression severity measure. Journal of General Internal Medicine. 2001;16:606-13.

40. Spitzer RL, Kroenke K, Williams JB, Lowe B. A brief measure for assessing generalized anxiety disorder: the GAD-7. Archives of Internal Medicine. 2006;166:1092-7.

41. Foa EB, Huppert JD, Leiberg S, Langner R, Kichic R, Hajcak G, et al. The Obsessive-Compulsive Inventory: development and validation of a short version. Psychological Assessment. 2002;14:485-96.

42. Moran P, Leese M, Lee T, Walters P, Thornicroft G, Mann A. Standardised Assessment of Personality - Abbreviated Scale (SAPAS): preliminary validation of a brief screen for personality disorder. Brritish Journal of Psychiatry. 2003;183:228-32.

43. Tyrer P, Morgan J, Cicchetti D. The Dependent Personality Questionnaire (DPQ): a screening instrument for dependent personality. International Journal of Social Psychiatry. 2004;50:10-7.

44. Dawson DA, Grant BF, Stinson FS, Zhou Y. Effectiveness of the derived alcohol use disorders identification test (AUDIT-C) in screening for alcohol use disorders and risk drinking in the US general population. Alcoholism: Clinical and Experimental Research. 2005;29:844-54.

45. Smith PC, Schmidt SM, Allensworth-Davies D, Saitz R. A single-question screening test for drug use in primary care. Archives of Internal Medicine. 2010;170:1155-60.

46. Borschmann R, Barrett B, Hellier JM, Byford S, Henderson C, Rose D, et al. Joint crisis plans for people with borderline personality disorder: feasibility and outcomes in a randomised controlled trial. The British Journal of Psychiatry. 2013;202:357-64.

47. Harris R. The Happiness Trap: Stop Struggling, Start Living. London: Robinson; 2008.

48. Kemp R. Face COVID: How to respond effectively to the Corona crisis. London: Central and North West London NHS Foundation Trust; 2020.

49. Tyrer H. Tackling Health Anxiety: A CBT Handbook. Cambridge: Cambridge University Press; 2013.

For peer review only - http://bmjopen.bmj.com/site/about/guidelines.xhtml

50. Billingham SA, Whitehead AL, Julious SA. An audit of sample sizes for pilot and feasibility trials being undertaken in the United Kingdom registered in the United Kingdom Clinical Research Network database. BMC Medical Research Methodology. 2013;13:104.

51. Dhand NK, Khatkar MS. Statulator: An online statistical calculator. Sample Size Calculator for Estimating a Single Proportion. 2014.

52. Avery KNL, Williamson PR, Gamble C, O'Connell Francischetto E, Metcalfe C, Davidson P, et al. Informing efficient randomised controlled trials: exploration of challenges in developing progression criteria for internal pilot studies. BMJ Open. 2017;7:e013537.

53. Herbert E, Julious SA, Goodacre S. Progression criteria in trials with an internal pilot: an audit of publicly funded randomised controlled trials. Trials. 2019;20:493.

54. StataCorp. Stata Statistical Software: Release 17. College Station, TX: StataCorp LLC, 2021.

55. Hensen B, Mackworth-Young C, Simwinga M, Abdelmagid N, Banda J, Mavodza C, et al. Remote data collection for public health research in a COVID-19 era: ethical implications, challenges and opportunities. Health Policy and Planning. 2021;36:360-8.

For peer review only

For peer review only - http://bmjopen.bmj.com/site/about/guidelines.xhtml

Table 1: Study Assessment Schedule

Assessments	Screening	Baseline	3-month follow-up	6-month follow-up
Coronavirus Anxiety Scale	X	-	X	X
Single item psychosis history	X			
Demographic and clinical data (Age, gender, ethnicity, household composition, occupational status, medical history)	-	X	-	-
Standardised Assessment of Personality – Abbreviated Scale	-	X	-	-
Dependent Personality Questionnaire	-	X	-	X
Use of alcohol and drugs (AUDIT-C and the single-question screening test for drug use)	-	X	X	X
Generalised Anxiety Disorder 7-item scale	-	X	X	X
Short Health Anxiety Inventory	-	X	X	X
Patient Health Questionnaire-9	-	X	X	X
Work and Social Adjustment Scale	-	X	X	X
Behaviours intended to reduce the risk of exposure to COVID-19	-	X	X	X
Obsessive -Compulsive Inventory	-	X	X	X
Health-related quality of life (EQ-5D-3L)	-	X	X	X
Resource use (ADSUS)	-	X	X	X
Serious Adverse Events	-	X	X	X
Vaccination status	-	-	-	X
Trial participants only: number and length of therapy sessions received	-	-	-	X

For peer review only - http://bmjopen.bmj.com/site/about/guidelines.xhtml

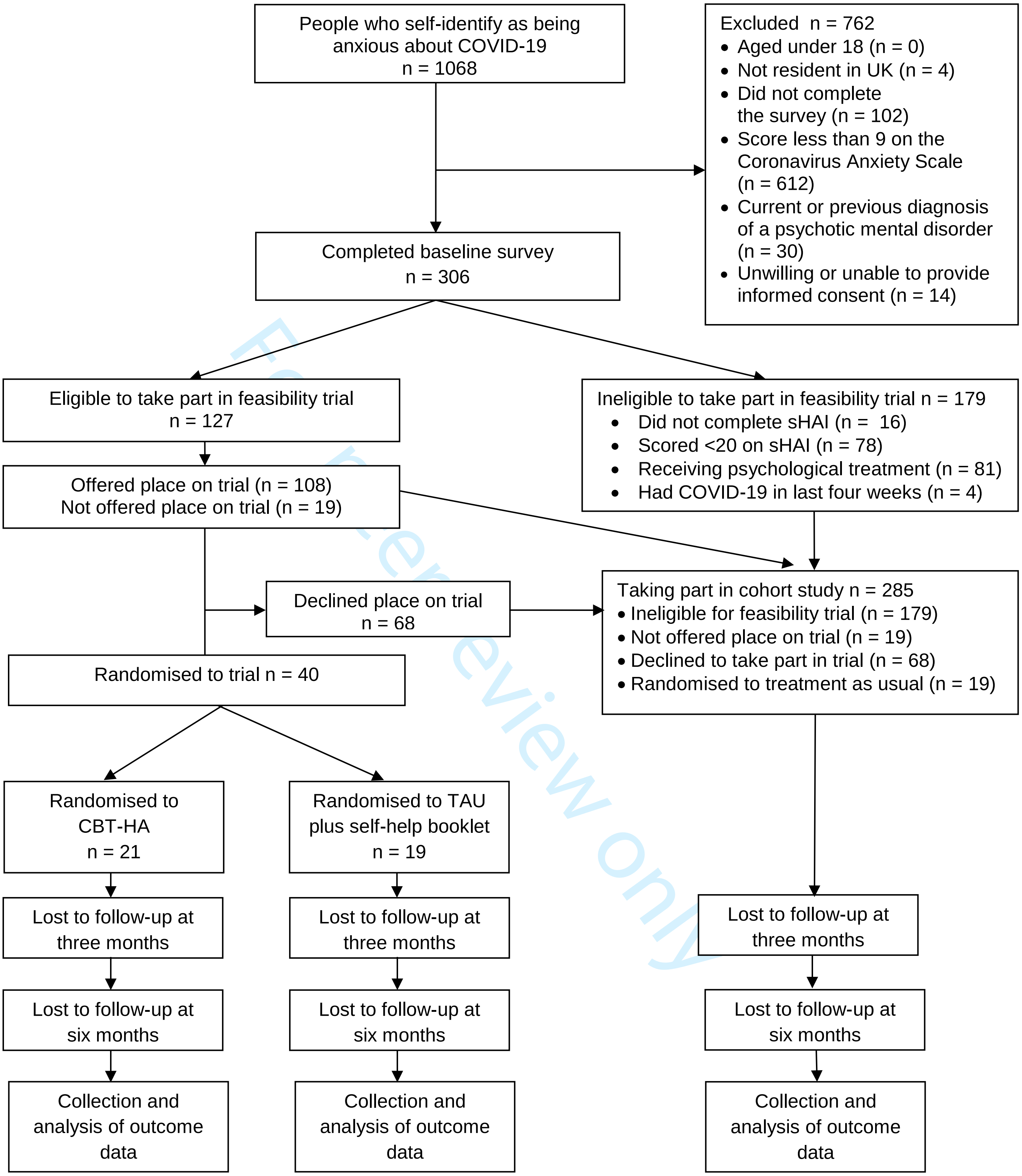

CBT-HA = Cognitive Behaviour Therapy for health anxiety. sHAI = Short for of Health Anxiety Inventory. TAU = Treatment as usual

Figure 1. Study flow diagram

For peer review only - http://bmjopen.bmj.com/site/about/guidelines.xhtml

SPIRIT 2013 Checklist: Recommended items to address in a clinical trial protocol and related documents*

Section/item	Item No	Description	Page number
**Administrative information**			
Title	1	Descriptive title identifying the study design, population, interventions, and, if applicable, trial acronym	1
Trial registration	2a	Trial identifier and registry name. If not yet registered, name of intended registry	2
	2b	All items from the World Health Organization Trial Registration Data Set	throughout
Protocol version	3	Date and version identifier	
Funding	4	Sources and types of financial, material, and other support	14
Roles and responsibilities	5a	Names, affiliations, and roles of protocol contributors	14
	5b	Name and contact information for the trial sponsor	5
	5c	Role of study sponsor and funders, if any, in study design; collection, management, analysis, and interpretation of data; writing of the report; and the decision to submit the report for publication, including whether they will have ultimate authority over any of these activities	14
	5d	Composition, roles, and responsibilities of the coordinating centre, steering committee, endpoint adjudication committee, data management team, and other individuals or groups overseeing the trial, if applicable (see Item 21a for data monitoring committee)	14
**Introduction**			
Background and rationale	6a	Description of research question and justification for undertaking the trial, including summary of relevant studies (published and unpublished) examining benefits and harms for each intervention	3-4
	6b	Explanation for choice of comparators	9-10
Objectives	7	Specific objectives or hypotheses	4

For peer review only - http://bmjopen.bmj.com/site/about/guidelines.xhtml

Trial design	8	Description of trial design including type of trial (eg, parallel group, crossover, factorial, single group), allocation ratio, and framework (eg, superiority, equivalence, noninferiority, exploratory)	4
**Methods: Participants, interventions, and outcomes**			
Study setting	9	Description of study settings (eg, community clinic, academic hospital) and list of countries where data will be collected. Reference to where list of study sites can be obtained	5
Eligibility criteria	10	Inclusion and exclusion criteria for participants. If applicable, eligibility criteria for study centres and individuals who will perform the interventions (eg, surgeons, psychotherapists)	5
Interventions	11a	Interventions for each group with sufficient detail to allow replication, including how and when they will be administered	9-10
	11b	Criteria for discontinuing or modifying allocated interventions for a given trial participant (eg, drug dose change in response to harms, participant request, or improving/worsening disease)	Not applicable
	11c	Strategies to improve adherence to intervention protocols, and any procedures for monitoring adherence (eg, drug tablet return, laboratory tests)	Not applicable
	11d	Relevant concomitant care and interventions that are permitted or prohibited during the trial	9
Outcomes	12	Primary, secondary, and other outcomes, including the specific measurement variable (eg, systolic blood pressure), analysis metric (eg, change from baseline, final value, time to event), method of aggregation (eg, median, proportion), and time point for each outcome. Explanation of the clinical relevance of chosen efficacy and harm outcomes is strongly recommended	7-8, 11
Participant timeline	13	Time schedule of enrolment, interventions (including any run-ins and washouts), assessments, and visits for participants. A schematic diagram is highly recommended (see Figure)	20
Sample size	14	Estimated number of participants needed to achieve study objectives and how it was determined, including clinical and statistical assumptions supporting any sample size calculations	10

Recruitment	15	Strategies for achieving adequate participant enrolment to reach target sample size	5-6
**Methods: Assignment of interventions (for controlled trials)**			
Allocation:			
Sequence generation	16a	Method of generating the allocation sequence (eg, computer-generated random numbers), and list of any factors for stratification. To reduce predictability of a random sequence, details of any planned restriction (eg, blocking) should be provided in a separate document that is unavailable to those who enrol participants or assign interventions	7
Allocation concealment mechanism	16b	Mechanism of implementing the allocation sequence (eg, central telephone; sequentially numbered, opaque, sealed envelopes), describing any steps to conceal the sequence until interventions are assigned	7
Implementatio n	16c	Who will generate the allocation sequence, who will enrol participants, and who will assign participants to interventions	7
Blinding (masking)	17a	Who will be blinded after assignment to interventions (eg, trial participants, care providers, outcome assessors, data analysts), and how	6-7
	17b	If blinded, circumstances under which unblinding is permissible, and procedure for revealing a participant's allocated intervention during the trial	Not applicable
**Methods: Data collection, management, and analysis**			
Data collection methods	18a	Plans for assessment and collection of outcome, baseline, and other trial data, including any related processes to promote data quality (eg, duplicate measurements, training of assessors) and a description of study instruments (eg, questionnaires, laboratory tests) along with their reliability and validity, if known. Reference to where data collection forms can be found, if not in the protocol	8
	18b	Plans to promote participant retention and complete follow-up, including list of any outcome data to be collected for participants who discontinue or deviate from intervention protocols	8

Data management	19	Plans for data entry, coding, security, and storage, including any related processes to promote data quality (eg, double data entry; range checks for data values). Reference to where details of data management procedures can be found, if not in the protocol	12-13
Statistical methods	20a	Statistical methods for analysing primary and secondary outcomes. Reference to where other details of the statistical analysis plan can be found, if not in the protocol	10-11
	20b	Methods for any additional analyses (eg, subgroup and adjusted analyses)	11
	20c	Definition of analysis population relating to protocol non-adherence (eg, as randomised analysis), and any statistical methods to handle missing data (eg, multiple imputation)	10-11
**Methods: Monitoring**			
Data monitoring	21a	Composition of data monitoring committee (DMC); summary of its role and reporting structure; statement of whether it is independent from the sponsor and competing interests; and reference to where further details about its charter can be found, if not in the protocol. Alternatively, an explanation of why a DMC is not needed	14
	21b	Description of any interim analyses and stopping guidelines, including who will have access to these interim results and make the final decision to terminate the trial	Not applicable
Harms	22	Plans for collecting, assessing, reporting, and managing solicited and spontaneously reported adverse events and other unintended effects of trial interventions or trial conduct	8-9
Auditing	23	Frequency and procedures for auditing trial conduct, if any, and whether the process will be independent from investigators and the sponsor	12
**Ethics and dissemination**			
Research ethics approval	24	Plans for seeking research ethics committee/institutional review board (REC/IRB) approval	1, 11-12

For peer review only - http://bmjopen.bmj.com/site/about/guidelines.xhtml

Protocol amendments	25	Plans for communicating important protocol modifications (eg, changes to eligibility criteria, outcomes, analyses) to relevant parties (eg, investigators, REC/IRBs, trial participants, trial registries, journals, regulators)	Not applicable
Consent or assent	26a	Who will obtain informed consent or assent from potential trial participants or authorised surrogates, and how (see Item 32)	11-12
	26b	Additional consent provisions for collection and use of participant data and biological specimens in ancillary studies, if applicable	Not applicable
Confidentiality	27	How personal information about potential and enrolled participants will be collected, shared, and maintained in order to protect confidentiality before, during, and after the trial	12
Declaration of interests	28	Financial and other competing interests for principal investigators for the overall trial and each study site	14
Access to data	29	Statement of who will have access to the final trial dataset, and disclosure of contractual agreements that limit such access for investigators	14
Ancillary and post-trial care	30	Provisions, if any, for ancillary and post-trial care, and for compensation to those who suffer harm from trial participation	Not applicable
Dissemination policy	31a	Plans for investigators and sponsor to communicate trial results to participants, healthcare professionals, the public, and other relevant groups (eg, via publication, reporting in results databases, or other data sharing arrangements), including any publication restrictions	12-13
	31b	Authorship eligibility guidelines and any intended use of professional writers	Not applicable
	31c	Plans, if any, for granting public access to the full protocol, participant-level dataset, and statistical code	Not applicable
**Appendices**			
Informed consent materials	32	Model consent form and other related documentation given to participants and authorised surrogates	Available on request
Biological specimens	33	Plans for collection, laboratory evaluation, and storage of biological specimens for genetic or molecular analysis in the current trial and for future use in ancillary studies, if applicable	Not applicable

For peer review only

*It is strongly recommended that this checklist be read in conjunction with the SPIRIT 2013 Explanation & Elaboration for important clarification on the items. Amendments to the protocol should be tracked and dated. The SPIRIT checklist is copyrighted by the SPIRIT Group under the Creative Commons "Attribution-NonCommercial-NoDerivs 3.0 Unported" license.

For peer review only

Severe COVID anxiety among adults in the United Kingdom: protocol for a cohort study and nested feasibility trial of modified Cognitive Behaviour Therapy for Health Anxiety.

Journal:	*BMJ Open*
Manuscript ID	bmjopen-2021-059321.R2
Article Type:	Protocol
Date Submitted by the Author:	16-Aug-2022
Complete List of Authors:	Crawford, Mike; Imperial College London, Psychological Medicine<br>Leeson, Verity; Imperial College London<br>McQuaid, Aisling; Imperial College London<br>Samuel, Oluwaseun; Imperial College London<br>King, Jacob D; Imperial College London<br>Di Simplicio, Martina; Imperial College London<br>Tyrer, Peter; Imperial College London, Psychological medicine<br>Tyrer, Helen; Imperial College London<br>Watt, Richard; University College London, Epidemiology and Public Health<br>Barnicot, Kirsten; City University of London
**Primary Subject Heading**:	Mental health
Secondary Subject Heading:	Mental health
Keywords:	COVID-19, Anxiety disorders < PSYCHIATRY, Clinical trials < THERAPEUTICS

Severe COVID anxiety among adults in the United Kingdom: protocol for a cohort study and nested feasibility trial of modified Cognitive Behaviour Therapy for Health Anxiety.

Mike J Crawford*, Verity C Leeson, Aisling McQuaid, Oluwaseun Samuel, Jacob D King, Martina Di Simplicio, Peter Tyrer, Helen Tyrer, Richard G Watt, Kirsten Barnicot.

* Corresponding author

Mike J Crawford. Professor of Mental Health Research, Imperial College London

m.crawford@imperial.ac.uk

For peer review only

ABSTRACT

Introduction

Some people are so anxious about COVID that it impairs their functioning. However, little is known about the course of severe COVID anxiety or what can be done to help people who experience it.

Methods and analysis

Cohort study with a nested feasibility trial with follow-up at three and six months. We recruited 306 people who were aged 18 and over, lived in the United Kingdom and had severe COVID anxiety (indicated by a score of nine or more on the Coronavirus Anxiety Scale). To take part in the nested feasibility trial, participants also had to have a score of 20 or more on the Short Health Anxiety Inventory. We excluded people from the trial if they had had COVID-19 within the previous four weeks, if they were currently self-isolating or if they were already receiving psychological treatment.

We publicised the study nationally through adverts, social media and posts on chat boards. We also recruited participants via clinicians working in primary and secondary care NHS services in London. All those in the active arm will be offered five to ten sessions of remotely delivered modified Cognitive Behaviour Therapy for Health Anxiety (CBT-HA). We will examine the proportion of participants who remain above threshold on the Coronavirus Anxiety Scale at three and six months and factors that influence levels of COVID anxiety over

For peer review only - http://bmjopen.bmj.com/site/about/guidelines.xhtml

six months using mixed-effects logistic regression. The key feasibility metrics for the nested trial are the level of uptake of CBT-HA and the rate of follow-up.

Ethics and dissemination

Approved by Leicester Central Research Ethics Committee (reference: 20/EM/0238). The results of the study will be published in peer-reviewed scientific journals.

Trial registration: International Standard Randomised Control Trial Number Register - ISRCTN14973494

For peer review only

Strengths of the study

- The first study to explore the feasibility of using a modified form of CBT-HA for people with severe COVID anxiety.
- Use of remote methods for data collection and delivering the intervention, which enable participation by those who are unable to attend face-to-face meetings.

Limitations of the study

- The study design means that we will not be able to estimate the prevalence of severe COVID anxiety in the general population.
- Reliance on remote methods for recruitment and follow-up may impact on the follow up rate.

INTRODUCTION

The COVID-19 pandemic is having a major impact on mental health throughout the world.(1, 2) In the UK, there was a marked increase in the proportion of people experiencing anxiety after the first wave of the pandemic,(3-5) a trend which has also been seen in many other countries.(6) Evidence from longitudinal studies in the UK suggest that a range of factors including social isolation, job insecurity and financial problems were important.(3, 7) Qualitative data collected during his period reinforce the importance of these factors and

For peer review only - http://bmjopen.bmj.com/site/about/guidelines.xhtml

have also highlighted disruption to work and other routines and a sense of lack of control.(8, 9)

Fear is an appropriate response to a new infectious disease like COVID-19 that threatens health, lives and livelihoods. At the start of the pandemic it was unclear how many people were likely to become infected, what the case fatality rate was and to what extent people who survived the initial illness would go on to experience long-term negative effects. Under these circumstances, fearfulness and anxiety are to be expected. Indeed, research conducted in the early phase of the pandemic found that fear of COVID-19 can be adaptive, resulting in greater adherence to public health measures aimed at limiting the spread of the virus.(10, 11) However, as the pandemic has progressed it has become clear that some people have become so anxious about COVID that it is impacting on their mental health and social functioning. People with severe COVID anxiety are more likely to report harmful use of alcohol and drugs, hopelessness and suicidal thoughts.(12, 13) To date, researchers investigating this topic have used slightly different definitions of 'COVID anxiety'. One of the first teams to investigate the subject, led by Lee and colleagues in Virginia (USA), used a narrow definition based on the cognitive, behavioural and physiological components of anxiety.(12-13) More recently teams such as Spada and colleagues in London (UK), have adopted a broader definition that incorporates a wider range of behavioural responses including avoidance, checking and threat monitoring.(14, 15) Nonetheless, all those investigating COVID anxiety agree that core symptoms are those found in other anxiety disorders, and it impacts on behaviour and social functioning.

At the start of the pandemic it was unclear what could be done to help people who appeared to have severe COVID anxiety. In the absence of evidence, governments, professional organisations and mental health services recommended general public mental health measures, such as maintaining contact with family and friends, keeping regular routines and sleeping times, and avoiding drug use and excessive use of alcohol.(16, 17). However, in our discussions with patients referred to mental health services who had severe COVID anxiety, we found that some appeared to be suffering from health anxiety. Health anxiety, formerly referred to as hypochondriasis, is a condition in which people become overwhelmed by fears of becoming ill.(18)

For peer review only - http://bmjopen.bmj.com/site/about/guidelines.xhtml

Health anxiety was a major contributor to poor mental health during previous pandemics.(19, 20) Emerging evidence from public surveys in Europe, Asia and North America have shown it is also contributing to poor mental health during the COVID-19 pandemic.(21-23) People with health anxiety may repeatedly search the internet and other media for information about health conditions, leading to mental distress and impaired functioning.(24, 25) People with health anxiety may misinterpret bodily sensations as symptoms of a physical illness and seek reassurance from others.(26) However, symptom monitoring and reassurance seeking may increase the amount of time that people think about being unwell and increase their level of anxiety.(18) During the COVID pandemic people may be at greater risk of health anxiety because of widespread media coverage, misinformation about COVID and uncertainty about the course of the pandemic.(27) In addition to this people are being actively encouraged to stay alert for signs of infection and get tested for COVID-19 as soon as possible if even mild signs of infection occur. This approach is an important public health strategy for limiting the transmission of COVID,(28) but it may also increase the likelihood of people who are predisposed to being anxious about their health becoming severely anxious to the extent that reduces their quality of life and impacts on their ability to function.(20)

The link between health anxiety and poor mental health seen in previous pandemics, is important because health anxiety is treatable. Cognitive Behavioural Therapy for Health Anxiety (CBT-HA) leads to long-term reductions in levels of health anxiety, generalised anxiety and depression.(29, 30) In recent years the intervention has been successfully modified to enable it to be delivered remotely.(31)

As social distancing measures are relaxed and people are able to resume social and occupational roles, it is essential to understand the course and consequences of severe COVID anxiety and examine interventions that could improve people's mental health and functioning. However, at this stage, we do not know the course of COVID anxiety or the contribution that health anxiety plays in maintaining it. While CBT-HA is effective under normal circumstances, we need to find out if it is an appropriate intervention to offer during a global pandemic, when people continue to be asked to be vigilant in spotting the signs of infection in an effort to prevent transmission of the virus.

For peer review only - http://bmjopen.bmj.com/site/about/guidelines.xhtml

Objectives

This study aims to reduce current gaps in knowledge and contribute to the development of a more effective response to health anxiety during pandemics. The study objectives are to;

1) Assess the impact of severe COVID anxiety on people's social functioning and health-related quality of life,

2) Examine the course of severe COVID anxiety over a six-month period,

3) Identify factors that influence the course of severe COVID anxiety, and

4) Test the feasibility of a randomised controlled trial of CBT-HA for people with severe COVID anxiety and health anxiety aimed at improving mental health and social functioning.

METHODS AND ANALYSIS

The COVID Anxiety Project is a cohort study with a nested feasibility trial. People taking part in the study will be followed up for six months. The trial is a researcher-blind, parallel-arm, randomised controlled feasibility trial, which is compliant with Standard Protocol Items: Recommendations for Interventional Trials guidelines.(32)

Study population and sample

Study participants were recruited from the general public in the United Kingdom and from those in contact with primary care and secondary care mental health services in London. We aimed to recruit people who self-identified as being anxious about COVID-19 using a broad range of methods including social media sites (Facebook, Reddit, Instagram and Twitter) and mental health charities (MQ research and Anxiety UK). We publicised the study via 19 primary care practices in north and west London and through colleagues working in secondary care mental health services in Central and North West London NHS Foundation Trust, City and East London NHS Foundation Trust and West London NHS Trust.

To be eligible to take part in the cohort study, potential participants had to be aged 18 or over, live in the United Kingdom and score nine or more on the Coronavirus Anxiety Scale.(12) We used a threshold of nine or more on the CAS because this identifies those with moderate or severe functional impairment with 90% sensitivity, 85% specificity, and a false positive rate of 15%.(12)

We excluded people who had a current or previous diagnosis of a psychotic mental disorder. To take part in the nested feasibility trial, participants also had to have a score of 20 or more

For peer review only - http://bmjopen.bmj.com/site/about/guidelines.xhtml

on the Short Health Anxiety Inventory (SHAI).(33) We excluded people from the feasibility trial if, at the time of the baseline assessment, they:

- had had COVID-19 in the prior four weeks (defined as a positive antigen test or a diagnosis by a clinician),
- were self-isolating on the advice of a doctor or the NHS Test and Trace service; or
- were already receiving psychological treatment for any condition.

The cohort study will be made up of all study participants with the exception of those offered CBT-HA as part of the feasibility trial.

Recruitment and follow-up

Participant flow through the study is presented in figure 1. We posted adverts on social media sites and websites of mental health charities and asked staff working in primary and secondary care NHS services to direct potential participants to a dedicated study website that was hosted by Qualtrics (www.qualtrics.com). The site included a copy of the Participant Information Sheet. Potential participants were asked to sign and date an online consent form, confirm their eligibility to take part in the study, and complete the Coronavirus Anxiety Scale (see supplementary file 1). The survey was designed so that it could only be completed once from any one IP address.(34) Those who scored nine or more on the scale and met other eligibility criteria were invited to complete the baseline survey. Those who were not eligible for the study were directed to a webpage which listed sources of mental health support.

We identified potential participants for the feasibility trial from their responses to the baseline survey. We aimed to recruit participants according to the capacity of study therapists to deliver CBT-HA to those in the active arm of the trial. When therapists had capacity to work with more people, we emailed those who recently joined the cohort study and met the additional eligibility criteria a copy of an additional Participant Information Sheet and directed them to an online consent form (see supplementary file 2). A researcher contacted these participants by telephone to answer any queries they had about the study, confirm that they met study eligibility criteria, support them to complete the online consent form and counter sign it. The researcher then contacted the trial manager who randomised the participant. Any participant who was approached to take part in the randomised trial and was ineligible or declined to take part, remained in the cohort study and will be asked to complete subsequent follow-up surveys.

For peer review only - http://bmjopen.bmj.com/site/about/guidelines.xhtml

Following attempts by what appeared to be automated survey-takers or 'bots' to take part in the study, we added a CAPTCHA question and a 'trap' question.(34, 35) The 'trap' question provided a range of valid and non-valid but plausible options for how the potential participant had heard about the study, with only those providing a valid response being invited to complete the baseline survey.

Between February 2021 and September 2021, we recruited 306 participants to the study. Of these, 127 (41.5%) met inclusion criteria for the feasibility trial. Among 108 participants offered a place on the trial 40 (37.0%) accepted and were randomised. The total sample for the cohort study is 285 comprising 179 who were ineligible for the trial, 19 who were eligible but not offered a place due to limited capacity of therapists to take on new patients, 68 who were offered a place on the trial but declined it and 19 people who were randomised to treatment as usual.

All participants in the cohort study and the feasibility trial will be sent an automated email to ask them to complete the three- and six-month follow-up survey. Those who have not responded after one week will be sent reminders. Trial participants who do not complete follow-up interviews will also be contacted by researchers by email (or telephone if contact number is available) with requests to complete the follow-up surveys.

Randomisation and blinding

We generated a randomisation list using the independent web-based service 'sealed envelope' (https://www.sealedenvelope.com/simple-randomiser/v1/lists). We stratified according to score on the short Health Anxiety Inventory (≤24 and ≥25) and the Dependent Personality Questionnaire (≤11 and ≥12) using a ratio of CBT-HA to control treatment of 1:1. We included the Dependent Personality Questionnaire as a stratification variable because previous research has demonstrated that the presence of dependent personality traits may influence the uptake and impact of CBT-HA.(36)

Throughout the study, the randomisation list will be encrypted and stored electronically by the trial manager. At the end of the study the randomisation list will be unencrypted and placed in the Trial Master File. The trial manager generated the allocation of each new trial participant by allocating them to the next treatment arm in the predetermined list. The participant was informed of their allocation by telephone or email, and the first therapy session scheduled for those allocated to CBT-HA. Researchers will have very little contact with participants other than to encourage them to complete follow up surveys. In the

For peer review only - http://bmjopen.bmj.com/site/about/guidelines.xhtml

unlikely event that researchers speak to a study participant after they have been randomised, they will start by reminding them of the need to make sure they are not aware of whether the participant was offered CBT-HA or control treatment.

Assessments

The timing and sequence of all assessments are summarised in table 1.

Table 1: Study Assessment Schedule

Assessments	Screening	Baseline	3-month follow-up	6-month follow-up
Coronavirus Anxiety Scale	X	-	X	X
Single item psychosis history	X			
Demographic and clinical data (Age, gender, ethnicity, household composition, occupational status, medical history)	-	X	-	-
Standardised Assessment of Personality – Abbreviated Scale	-	X	-	-
Dependent Personality Questionnaire	-	X	-	X
Use of alcohol and drugs (AUDIT-C and the single-question screening test for drug use)	-	X	X	X
Generalised Anxiety Disorder 7-item scale	-	X	X	X
Short Health Anxiety Inventory	-	X	X	X
Patient Health Questionnaire-9	-	X	X	X
Work and Social Adjustment Scale	-	X	X	X
Behaviours intended to reduce the risk of exposure to COVID-19	-	X	X	X
Obsessive -Compulsive Inventory	-	X	X	X
Health-related quality of life (EQ-5D-3L)	-	X	X	X
Resource use (ADSUS)	-	X	X	X
Serious Adverse Events	-	X	X	X
Vaccination status	-	-	-	X

For peer review only - http://bmjopen.bmj.com/site/about/guidelines.xhtml

Trial participants only: number and length of therapy sessions received	-	-	-	X

Screening assessment

To take part in the cohort study, potential participants had to score nine or more on the Coronavirus Anxiety Scale (CAS).(12, 13) Developed by Sherman Lee in the USA, the scale was designed to provide a brief and reliable measure of the level of COVID-related anxiety that people experience. It comprises five questions on the frequency of anxious thoughts, somatic symptoms and sleep disturbance triggered by reading, thinking or hearing about COVID.

Baseline assessment and covariates

At baseline we collected self-reported data on demographic factors (age, gender, ethnicity, household composition, occupational status), physical health (current medical conditions), and exposure to COVID (whether they had had COVID, whether they had been admitted to hospital with COVID). We asked participants whether they live with or care for someone that might get seriously ill if infected with COVID because of their health or age and whether someone in their family or a close friend had been in hospital with COVID.

We also asked participants questions about behaviours intended to reduce the risk of exposure to COVID. These questions were developed with the help of the members of the Lived Experience Advisory Panel and covered five behaviours which people had used or heard of others using in an effort to avoid catching COVID: staying at home, avoiding shops, washing or discarding letters and parcels, increased handwashing and increased washing of clothes. People who lived with school age children were also asked whether they had stopped them attending school because of concerns about COVID.

We assessed self-reported functioning using the Work and Social Adjustment scale (WSAS)(37) and health-related quality of life using the EQ-5D-3L.(38) We assessed mental health using the Patient Health Questionnaire-9,(39) Generalised Anxiety Disorder 7-item scale,(40) Obsessive -Compulsive Inventory -Revised,(41) the short form of the Health Anxiety Inventory (SHAI),(33) the Standardised Assessment of Personality – Abbreviated Scale,(42) the Dependent Personality Questionnaire,(43) use of alcohol (AUDIT-C)(44) and a single-question screening test for drug use.(45) Finally, we asked participants to indicate

For peer review only - http://bmjopen.bmj.com/site/about/guidelines.xhtml

admissions to hospital, contacts with primary and secondary care services using items from the Adult Service User Schedule.(46)

Follow-up surveys

All those taking part in the cohort study or the feasibility trial are asked to complete follow-up surveys three and six months after the baseline survey. The content of these surveys was similar to the baseline survey, except that we did not repeat the personality assessments (SAPAS and DPQ), and we added a question at six months on whether people had received a COVID-19 vaccination (see table 1).

In recognition of their time and support, all those who completed a baseline interview were sent a £10 gift voucher and a further £20 voucher will be offered to those that complete the six-month follow-up interview.

Serious adverse events

Adverse events that are identified in the survey (e.g. worsening or newly emerging clinical phenomena) will be added to an adverse event log. Data on Serious Adverse Event (SAE) including death, hospitalisation and life-threatening events are recorded at baseline, and three and six month follow-up. Therapists will also be regularly reminded that they should report all serious adverse events to the clinical trial team. SAEs will be recorded on a non-CTIMP SAE form and forwarded within 24 hours to the Chief Investigator and sponsor if judged to be unexpected and related to the research procedures. The Research Ethics Committee will be notified within 15 days of the Chief Investigator becoming aware of any such SAE.

Interventions

All those who agree to take part in the feasibility trial will be sent a self-help booklet developed by staff at Central and North West London NHS Foundation Trust that is derived from the work of Russ Harris on Acceptance and Commitment Therapy,(47) and aims to help people cope with the COVID crisis by building resilience, identifying and implementing healthy coping strategies and general advice on health and wellbeing.(48) All participants will be able access treatment as usual, which includes access to NHS primary care services and referral on to specialist services if required.

All those in the active arm of the feasibility trial will be offered five to ten sessions of modified Cognitive Behavioural Therapy for Health Anxiety (CBT-HA) based on a published treatment manual.(49) Therapists will start by taking a detailed history of the person's

For peer review only - http://bmjopen.bmj.com/site/about/guidelines.xhtml

thoughts and fears about COVID and exploring their beliefs about the impact of COVID and their ability to cope, in order to develop a formulation. Therapists will seek to identify behaviours such as symptom monitoring and reassurance seeking that could be maintaining the person's anxiety. They will then use Socratic dialogue, diary keeping and behavioural experiments to help participants recognise the links between their thoughts and behaviour and explore ways to reduce their anxiety.

All CBT-HA sessions will be delivered by phone or videoconferencing, in accordance with the participant's preference. Sessions will generally last between 30 and 50 minutes and be offered on a weekly or fortnightly basis. Therapists will consider delaying their final session in order to give people an opportunity to use the techniques and skills they have learned and reinforce the changes they have made. Sessions will be delivered in keeping with a published treatment manual but modified to meet the difficulties that people in the study are experiencing. Sessions will be supplemented by booklets summarising the causes of health anxiety and how patients can begin to overcome their fears.

Each therapist will record the number and length of sessions they offer participants so that we can calculate the proportion of people who are offered CBT-HA who start and complete treatment and the number of sessions that people receive.

All therapists have a degree in a health-related subject and have previous experience of delivering psychological treatments. All therapists attended a 90-minute training session by Dr Helen Tyrer and will receive fortnightly supervision sessions delivered by Dr Tyrer.

The nature of modifications to the original CBT-HA model will be recorded during the study and presented alongside the results of the feasibility trial.

Sample size

We aimed to recruit sufficient numbers of participants to the cohort study in order to randomise 40 participants to the feasibility trial, which is a typical size for such a trial.(50) A sample of 40 participants would enable us to detect study consent and intervention completion rates of 50%, with 95% confidence intervals of +/-11% and +/- 15% respectively. Assuming a rate of follow-up at six months of 75%, our cohort of 285 study participants will generate valid data on 213 people. A sample of 196 participants in the cohort study will enable us to estimate a remission rate of 15% +/- 5%, with 95% confidence.(51)

Data analysis

For peer review only - http://bmjopen.bmj.com/site/about/guidelines.xhtml

We will start by examining the characteristics of the study sample, including the prevalence of coexisting physical and mental health conditions, levels of disturbance in work and social functioning, quality of life, and actions taken to try to avoid catching COVID using simple descriptive statistics. For the cohort study, we will examine the proportion who remain above threshold for severe COVID anxiety (9 or above on the CAS) and for COVID anxiety (5 or more on the CAS) at three and six months after completion of the baseline survey. We will examine factors that influence changes in CAS scores over this period using linear regression analysis. The analysis will be performed in two stages. Initially the separate association between each factor and CAS at each outcome timepoint will be assessed separately. CAS score at baseline will be included in all these analyses, as the inclusion of this factor will mean that the analyses reflect factors associated with change in CAS from baseline. The second stage of the analysis will examine the joint association between the factors and CAS score in a multivariable analysis. To restrict the number of variables in this stage of the analysis, only factors showing some association with the outcome ($p<0.2$) from the first stage of the analysis will be considered in the multivariable analyses. A selection procedure (e.g. backwards selection) will be considered to identify factors significantly associated with the outcome and to be included in the final model.

We anticipate that there will be little missing data because the platform we designed for data entry requires participants to complete every item of a questionnaire before moving on to the next section. However where the number of missing items on a given measure is 20% or less, then the missing value for the items will be substituted by the individual's mean score for the remaining items on the scale. If there are more than 20% missing items in the scale the outcome measure will not be calculated for the participant at that time point.

Our criteria for determining the success of the feasibility study, which are based on thresholds used in other recent feasibility and pilot studies,(52, 53) are: recruitment of at least 32 participants (80% of the target study sample of 40 participants), uptake of the intervention by at least 60% of participants in the active arm of the trial, and completion of follow-up interviews at six months by 75% of study participants. All data will be analysed in SPSS version 2.0 (SPSS Inc., Chicago IL) and Stata version 16.1.(54)

Patient and public involvement

For peer review only - http://bmjopen.bmj.com/site/about/guidelines.xhtml

Patients and the public contributed to the development of this proposal. Charlotte Green, who chaired the Patient & Carers Research Interest group at CNWL NHS Foundation Trust, helped develop the proposal and write the lay summary. We presented plans for the study to members of a local patient research interest group via Zoom. Members expressed strong support for the study and eight agreed to join a Lived Experience Advisory Group. Feedback from the group led to us to adding new questions on the impact of COVID anxiety. Members made suggestions for how to access potential participants, which we adopted. Members of the group also highlighted the importance of tailoring CBT-HA to the needs of individual patients, including making time for them to discuss broader concerns about their mental health and the impact of the pandemic. Members of the Lived Experience Advisory Group will help us prepare a plain English summary of study findings, which we will make available to all study participants and other members of the public through the study website.

ETHICS AND DISSEMINATION

Plans for the study were approved by Leicester Central Research Ethics Committee (reference: 20/EM/0238). The study will be conducted in accordance with the recommendations for physicians involved in research on human subjects adopted by the 18th World Medical Assembly, Helsinki 1964 and later revisions.

All research activity will be carried out after participants have given consent, and this documented on either an electronic or paper form. For the cohort study, the consent forms will be completed independently by the participant without researcher support but a member of the study team will be available to answer any questions prior to consent being given. Individual consent completed for the cohort study will be linked in Qualtrics to the questionnaires completed by the same participant at each timepoint in the cohort study to ensure that the person that gave consent is completing the assessments.

Separate consent to take part in the clinical trial will be completed by the participant during a telephone call with a member of the research team. The member of the research team that facilitates the completion of the consent process on the telephone will countersign the consent form to document that they were present.

Completed electronic consent forms will be downloaded from Qualtrics, printed and stored in a Master File. A copy of their completed consent form(s) will be provided to each

For peer review only - http://bmjopen.bmj.com/site/about/guidelines.xhtml

participant. Any original paper consent forms will also be stored in the Master File. All participants are free to withdraw at any time from the cohort study or clinical trial without giving reasons and without prejudicing further treatment.

The Chief Investigator will preserve the confidentiality of participants taking part in the study and is registered under the Data Protection Act. Data from the consent forms and questionnaires, including identifiable personal data, will be stored on Qualtrics, an online survey tool. Qualtrics has ISO 27001 certification, meaning that it meets the international standard for managing confidential and sensitive data. All data transfers to Imperial College London use TLS encryption. Imperial College will be the data controller and there is a service-level agreement in place between Imperial College and Qualtrics.

Where questionnaires are administered during a telephone interview rather than online, the researcher will type the responses into Qualtrics. Other personal data that is recorded during the study (e.g. therapy notes from CBT sessions) will be typed directly onto the secure server of Imperial College London and will be identified using only the participant identifier. Qualtrics allows data erasure and all study data will be deleted from Qualtrics at the end of the study, following extraction for analysis.

Paper documentation will be minimal during this research. An option to complete paper consent form and screening questionnaires was offered but not taken up by any participant.

The results of the study will be published in peer-reviewed scientific journals. We anticipate three key papers examining baseline data, follow-up data and the results of the feasibility trial. We will work with members of our Lived Experienced Advisory Panel to prepare a lay summary of findings from the study which we will post on the study website and will email all study participants the link to this summary.

DISCUSSION

This study aims to build on the results of previous research that has highlighted the impact of COVID anxiety on mental health during the current pandemic. By collecting prospective data on a sample of people who have high levels of COVID anxiety, we will examine its course over a six-month period. Having collected detailed information on demographic and clinical factors at baseline we will be able to examine the role these factors play in maintaining COVID anxiety over time. By conducting a nested feasibility trial we will be able

For peer review only - http://bmjopen.bmj.com/site/about/guidelines.xhtml

to examine the uptake and acceptability of an intervention for health anxiety, a condition which is strongly linked to anxiety during pandemics and has the potential to benefit people who experience these problems.

This study has a number of strengths and limitations. We believe that it is the first to examine the feasibility of offering people with severe COVID anxiety a psychological intervention for their distress. While many studies have examined the prevalence and aetiology of mental health problems during the COVID crisis, far fewer have started to explore the impact of interventions for mental health problems, and we are not aware of any studies examining the impact of CBT-HA for those who have coexisting health anxiety. By using remote methods for collecting study data and delivering the intervention, we have been able to include people in the study who were so anxious about COVID that they were unlikely to attend face-to-face appointments.

Because we did not use a general population sample for the survey, we will not be able to estimate the prevalence of severe COVID anxiety in the general population. Our reliance on remote means for data collection may have put off some people from taking part in the study. We did make provision for people taking part in the study via telephone interviews, telephone-based therapy and signing paper copies of consent forms. However, people with limited or no access to the internet may have been put off from taking part.

We are using the Coronavirus Anxiety Scale as our primary outcome measure.(12) Since the start of the study, other measures have been developed which assess a wider range of behaviours that are associated with COVID anxiety.(14) While we are also asking study participants to describe the frequency of avoidant behaviours associated with COVID anxiety, the psychometric properties of these questions have not be tested.

Our reliance on remote methods for recruitment and following up study participants may have limited our ability to develop a rapport with them and this could, in turn, affect our response rate.(55) We will keep in touch with study participants by email and offer people an honoraria to complete the follow-up surveys in an effort to minimise loss to follow up.

While the nested clinical trial is large enough to examine the feasibility of a future explanatory trial of CBT-HA for people with severe COVID anxiety it has not been powered to examine the clinical effectiveness of this intervention.

For peer review only - http://bmjopen.bmj.com/site/about/guidelines.xhtml

Figure 1. Study flow diagram

AUTHOR AFFILIATIONS

1) Professor Mike J Crawford (Corresponding author). Division of Psychiatry, Imperial College London, Commonwealth Building, Hammersmith Hospital Campus, Du Cane Road, London W12 0NN, UK. m.crawford@imperial.ac.uk
2) Dr Verity Leeson. Division of Psychiatry, Imperial College London, Commonwealth Building, Hammersmith Hospital Campus, Du Cane Road, London W12 0NN, UK. v.leeson@imperial.ac.uk
3) Ms Aisling McQuaid. Division of Psychiatry, Imperial College London, Commonwealth Building, Hammersmith Hospital Campus, Du Cane Road, London W12 0NN, UK. a.mc-quaid@imperial.ac.uk
4) Ms Oluwaseun Samuel. Division of Psychiatry, Imperial College London, Commonwealth Building, Hammersmith Hospital Campus, Du Cane Road, London W12 0NN, UK. o.samuel@imperial.ac.uk
5) Dr Jacob King. Division of Psychiatry, Imperial College London, Commonwealth Building, Hammersmith Hospital Campus, Du Cane Road, London W12 0NN, UK. j.king20@imperial.ac.uk
6) Dr Martina Di Simplicio. Division of Psychiatry, Imperial College London, Commonwealth Building, Hammersmith Hospital Campus, Du Cane Road, London W12 0NN, UK. m.di-simplicio@imperial.ac.uk
7) Professor Peter Tyrer. Division of Psychiatry, Imperial College London, Commonwealth Building, Hammersmith Hospital Campus, Du Cane Road, London W12 0NN, UK. p.tyrer@imperial.ac.uk
8) Dr Helen Tyrer, Division of Psychiatry, Imperial College London, Commonwealth Building, Hammersmith Hospital Campus, Du Cane Road, London W12 0NN, UK. htyrer07@gmail.com
9) Professor Richard G Watt Department of Epidemiology & Public Health, University College London, 1-19 Torrington Place, London, WC1E 7HB, UK. r.watt@ucl.ac.uk
10) Paul Bassett, Statsconsultancy Ltd, Amersham, Buckinghamshire, UK. paul@statsconsultancy.co.uk
11) Dr Kirsten Barnicot. Division of Health Services Research and Management, City, University of London, Northampton Square, London EC1V 0HB, UK. Kirsten.Barnicot@city.ac.uk

Authors' contributions

For peer review only - http://bmjopen.bmj.com/site/about/guidelines.xhtml

MC is the Chief Investigator who conceived the study and led the design of the study and the study protocol and preparation of this manuscript. VL helped design the study protocol, obtained study approvals and contributed to the preparation of this manuscript. AMcQ, OS and JK recruited study participants contributed to the design of the study and the study protocol and preparation of this manuscript. HT helped to develop the study intervention and contributed to the preparation of this manuscript. MdS, RW, PT and KB, contributed to the design of the study, the development of the study protocol and the preparation of this manuscript. JK and MC developed the statistical analysis plan. All authors read and approved the final manuscript.

Acknowledgements

We are grateful to the members of a Combined Independent Oversight Committee which will oversee project governance, data management and review safety data (Dr John Green (chair), Professor Khalida Ismail and Mr Robert Koch). We thank Paul Bassett, freelance statistican, for reviewing the protocol and revising a draft of the Statictical Analysis Plan. We also thank members of the lived experience reference group including Manisha Ahya, Anjie Chhapia, Charlotte Green, Sandra Jayacodi, Niruben Patel and Vikas Sharma.

Funding statement

This research is supported by funding from the NIHR Imperial Biomedical Research Centre (WBPP_PA2902), an National Institute for Health Research Health Technology Assessment Programme (16/157/02) and an NIHR Senior Investigator Application held by Professor Crawford (NF-SI-0515-10006). The views expressed are those of the authors and not necessarily those of the NIHR or the Department of Health and Social Care.The sponsor of the trial is Imperial College London.

COMPETING INTERESTS STATEMENT

Dr Helen Tyrer is author of a book on Cognitive Behaviour Therapy for treating people with Health Anxiety. Other authors declare that they have no competing interests.

For peer review only

REFERENCES

1. World Health Organization. Mental Health Considerations During COVID-19 Outbreak Geneva: World Health Organization, 2020.

2. Thombs BD, Bonardi O, Rice DB, Boruff JT, Azar M, He C, et al. Curating evidence on mental health during COVID-19: A living systematic review. Journal of Psychosomatic Research. 2020;133:110113.

3. Pierce M, Hope H, Ford T, Hatch S, Hotopf M, John A, et al. Mental health before and during the COVID-19 pandemic: a longitudinal probability sample survey of the UK population. The Lancet Psychiatry. 2020;7:883-92.

4. Jia R, Ayling K, Chalder T, Massey A, Broadbent E, Coupland C, et al. Mental health in the UK during the COVID-19 pandemic: cross-sectional analyses from a community cohort study. BMJ Open. 2020;10:e040620.

5. Santabarbara J, Lasheras I, Lipnicki DM, Bueno-Notivol J, Perez-Moreno M, Lopez-Anton R, et al. Prevalence of anxiety in the COVID-19 pandemic: An updated meta-analysis of community-based studies. Progress in Neuro-Psychopharmacology & Biological Psychiatry. 2021;109:110207.

6. Santomauro DF, Herrera AM, Shadid J, Zheng P, Ashbaugh C, Pigott DM, Abbafati C, Adolph C, Amlag JO, Aravkin AY, Bang-Jensen BL. Global prevalence and burden of depressive and anxiety disorders in 204 countries and territories in 2020 due to the COVID-19 pandemic. The Lancet. 2021;398:1700-12.

7. McPherson KE, McAloney-Kocaman K, McGlinchey E, Faeth P, Armour C. Longitudinal analysis of the UK COVID-19 Psychological Wellbeing Study: Trajectories of anxiety, depression and COVID-19-related stress symptomology. Psychiatry Research. 2021;304:114138.

8. Burton A, McKinlay A, Aughterson H, Fancourt D. Impact of the COVID-19 pandemic on the mental health and well-being of adults with mental health conditions in the UK: a qualitative interview study. Journal of Mental Health. 2021:1-8.

For peer review only - http://bmjopen.bmj.com/site/about/guidelines.xhtml

9. McKinlay AR, May T, Dawes J, Fancourt D, Burton A. 'You're just there, alone in your room with your thoughts': a qualitative study about the psychosocial impact of the COVID-19 pandemic among young people living in the UK. BMJ Open. 2022;12:e053676.

10. Harper CA, Satchell LP, Fido D, Latzman RD. Functional Fear Predicts Public Health Compliance in the COVID-19 Pandemic. International Journal of Mental Health and Addiction. 2021: 19:1875-88.

11. Chen SX, Ng JCK, Hui BPH, Au AKY, Wu WCH, Lam BCP, et al. Dual impacts of coronavirus anxiety on mental health in 35 societies. Scientific Reports. 2021;26:1-1.

12. Lee SA. Coronavirus Anxiety Scale: A brief mental health screener for COVID-19 related anxiety. Death Studies. 2020;44:393-401.

13. Lee SA, Mathis AA, Jobe MC, Pappalardo EA. Clinically significant fear and anxiety of COVID-19: A psychometric examination of the Coronavirus Anxiety Scale. Psychiatry Research. 2020;290:113112.

14. Nikcevic AV, Spada MM. The COVID-19 anxiety syndrome scale: Development and psychometric properties. Psychiatry Research. 2020;292:113322.

15. Albery IP, Spada MM, Nikcevic AV. The COVID-19 anxiety syndrome and selective attentional bias towards COVID-19-related stimuli in UK residents during the 2020-2021 pandemic. Clin Psychol Psychother. 2021;28:1367-78.

16. Department of Health and Social Care. New advice to support mental health during coronavirus outbreak London: Department of Health and Social Care; 2020 Available from: https://www.gov.uk/government/news/new-advice-to-support-mental-health-during-coronavirus-outbreak. Accessed 18/05/2022.

17. World Health Organization. Looking after our mental health Geneva: WHO; 2020. Available from: https://www.who.int/campaigns/connecting-the-world-to-combat-coronavirus/healthyathome/healthyathome---mental-health. Accessed 18/05/2022.

18. Tyrer P, Eilenberg T, Fink P, Hedman E, Tyrer H. Health anxiety: the silent, disabling epidemic. BMJ. 2016;353:i2250.

For peer review only - http://bmjopen.bmj.com/site/about/guidelines.xhtml

19. Wheaton MG, Abramowitz JS, Berman NC, Fabricant LE, Olatunji BO. Psychological predictors of anxiety in response to the H1N1 (swine flu) pandemic. doi:10.1007/s10608-011-9353-3. Cognitive Therapy and Research. 2012;36:210-8.

20. Asmundson GJG, Taylor S. How health anxiety influences responses to viral outbreaks like COVID-19: What all decision-makers, health authorities, and health care professionals need to know. Journal of Anxiety Disorders. 2020;71:102211.

21. Jungmann SM, Witthoft M. Health anxiety, cyberchondria, and coping in the current COVID-19 pandemic: Which factors are related to coronavirus anxiety? Journal of Anxiety Disorders. 2020;73:102239.

22. Nikcevic AV, Marino C, Kolubinski DC, Leach D, Spada MM. Modelling the contribution of the Big Five personality traits, health anxiety, and COVID-19 psychological distress to generalised anxiety and depressive symptoms during the COVID-19 pandemic. Journal of Affective Disorders. 2021;279:578-84.

23. Ozdin S, Bayrak Ozdin S. Levels and predictors of anxiety, depression and health anxiety during COVID-19 pandemic in Turkish society: The importance of gender. International Journal of Social Psychiatry. 2020;66:504-11.

24. Te Poel F, Baumgartner SE, Hartmann T, Tanis M. The curious case of cyberchondria: A longitudinal study on the reciprocal relationship between health anxiety and online health information seeking. Journal of Anxiety Disorders. 2016;43:32-40.

25. McMullan RD, Berle D, Arnaez S, Starcevic V. The relationships between health anxiety, online health information seeking, and cyberchondria: Systematic review and meta-analysis. Journal of Affective Disorders. 2019;245:270-8.

26. Olatunji BO, Deacon BJ, Abramowitz JS, Valentiner DP. Body vigilance in nonclinical and anxiety disorder samples: Structure, correlates, and prediction of health concerns. Behavior Therapy. 2007;38:392-401.

27. Tyrer P. COVID-19 health anxiety. World Psychiatry. 2020;19:307-8.

28. Girum T, Lentiro K, Geremew M, Migora B, Shewamare S. Global strategies and effectiveness for COVID-19 prevention through contact tracing, screening, quarantine, and isolation: a systematic review. Tropical Medicine and Health. 2020;48:1-5.

For peer review only - http://bmjopen.bmj.com/site/about/guidelines.xhtml

29. Tyrer P, Cooper S, Salkovskis P, Tyrer H, Crawford M, Byford S, et al. Clinical and cost-effectiveness of cognitive behaviour therapy for health anxiety in medical patients: a multicentre randomised controlled trial. The Lancet. 2014;383:219-25.

30. Cooper K, Gregory JD, Walker I, Lambe S, Salkovskis PM. Cognitive Behaviour Therapy for Health Anxiety: A Systematic Review and Meta-Analysis. Behavioural and Cognitive Psychotherapy. 2017;45:110-23.

31. Morriss R, Patel S, Malins S, Guo B, Higton F, James M, et al. Clinical and economic outcomes of remotely delivered cognitive behaviour therapy versus treatment as usual for repeat unscheduled care users with severe health anxiety: a multicentre randomised controlled trial. BMC Medicine. 2019;17:16.

32. Chan AW, Tetzlaff JM, Altman DG, Laupacis A, Gotzsche PC, Krleza-Jeric K, et al. SPIRIT 2013 statement: defining standard protocol items for clinical trials. Annuls of Internal Medicine. 2013;158:200-7.

33. Salkovskis PM, Rimes KA, Warwick HM, Clark DM. The Health Anxiety Inventory: development and validation of scales for the measurement of health anxiety and hypochondriasis. Psychological Medicine. 2002;32:843-53.

34. Storozuk A, Ashley M, Delage V, Maloney E. Got Bots? Practical Recommendations to Protect Online Survey Data from Bot Attacks. The Quantitative Methods for Psychology. 2020;16:472–81.

35. Liu M, Wronski L. Trap questions in online surveys: Results from three web survey experiments. International Journal of Market Research. 2018;60:32-49.

36. Sanatinia R, Wang D, Tyrer P, Tyrer H, Crawford M, Cooper S, et al. Impact of personality status on the outcomes and cost of cognitive-behavioural therapy for health anxiety. The British Journal of Psychiatry : the journal of mental science. 2016;209:244-50.

37. Mundt JC, Marks IM, Shear MK, Greist JH. The Work and Social Adjustment Scale: a simple measure of impairment in functioning. The British Journal of Psychiatry. 2002;180:461-4.

38. Brooks RG. EuroQoL- the current state of play. Health Policy. 1996;37:53-72.

For peer review only - http://bmjopen.bmj.com/site/about/guidelines.xhtml

39. Kroenke K, Spitzer RL, Williams JB. The PHQ-9: validity of a brief depression severity measure. Journal of General Internal Medicine. 2001;16:606-13.

40. Spitzer RL, Kroenke K, Williams JB, Lowe B. A brief measure for assessing generalized anxiety disorder: the GAD-7. Archives of Internal Medicine. 2006;166:1092-7.

41. Foa EB, Huppert JD, Leiberg S, Langner R, Kichic R, Hajcak G, et al. The Obsessive-Compulsive Inventory: development and validation of a short version. Psychological Assessment. 2002;14:485-96.

42. Moran P, Leese M, Lee T, Walters P, Thornicroft G, Mann A. Standardised Assessment of Personality - Abbreviated Scale (SAPAS): preliminary validation of a brief screen for personality disorder. Brritish Journal of Psychiatry. 2003;183:228-32.

43. Tyrer P, Morgan J, Cicchetti D. The Dependent Personality Questionnaire (DPQ): a screening instrument for dependent personality. International Journal of Social Psychiatry. 2004;50:10-7.

44. Dawson DA, Grant BF, Stinson FS, Zhou Y. Effectiveness of the derived alcohol use disorders identification test (AUDIT-C) in screening for alcohol use disorders and risk drinking in the US general population. Alcoholism: Clinical and Experimental Research. 2005;29:844-54.

45. Smith PC, Schmidt SM, Allensworth-Davies D, Saitz R. A single-question screening test for drug use in primary care. Archives of Internal Medicine. 2010;170:1155-60.

46. Borschmann R, Barrett B, Hellier JM, Byford S, Henderson C, Rose D, et al. Joint crisis plans for people with borderline personality disorder: feasibility and outcomes in a randomised controlled trial. The British Journal of Psychiatry. 2013;202:357-64.

47. Harris R. The Happiness Trap: Stop Struggling, Start Living. London: Robinson; 2008.

48. Kemp R. Face COVID: How to respond effectively to the Corona crisis. London: Central and North West London NHS Foundation Trust; 2020.

49. Tyrer H. Tackling Health Anxiety: A CBT Handbook. Cambridge: Cambridge University Press; 2013.

For peer review only - http://bmjopen.bmj.com/site/about/guidelines.xhtml

50. Billingham SA, Whitehead AL, Julious SA. An audit of sample sizes for pilot and feasibility trials being undertaken in the United Kingdom registered in the United Kingdom Clinical Research Network database. BMC Medical Research Methodology. 2013;13:104.

51. Dhand NK, Khatkar MS. Statulator: An online statistical calculator. Sample Size Calculator for Estimating a Single Proportion. 2014.

52. Avery KNL, Williamson PR, Gamble C, O'Connell Francischetto E, Metcalfe C, Davidson P, et al. Informing efficient randomised controlled trials: exploration of challenges in developing progression criteria for internal pilot studies. BMJ Open. 2017;7:e013537.

53. Herbert E, Julious SA, Goodacre S. Progression criteria in trials with an internal pilot: an audit of publicly funded randomised controlled trials. Trials. 2019;20:493.

54. StataCorp. Stata Statistical Software: Release 17. College Station, TX: StataCorp LLC, 2021.

55. Hensen B, Mackworth-Young C, Simwinga M, Abdelmagid N, Banda J, Mavodza C, et al. Remote data collection for public health research in a COVID-19 era: ethical implications, challenges and opportunities. Health Policy and Planning. 2021;36:360-8.

For peer review only

For peer review only

For peer review only - http://bmjopen.bmj.com/site/about/guidelines.xhtml

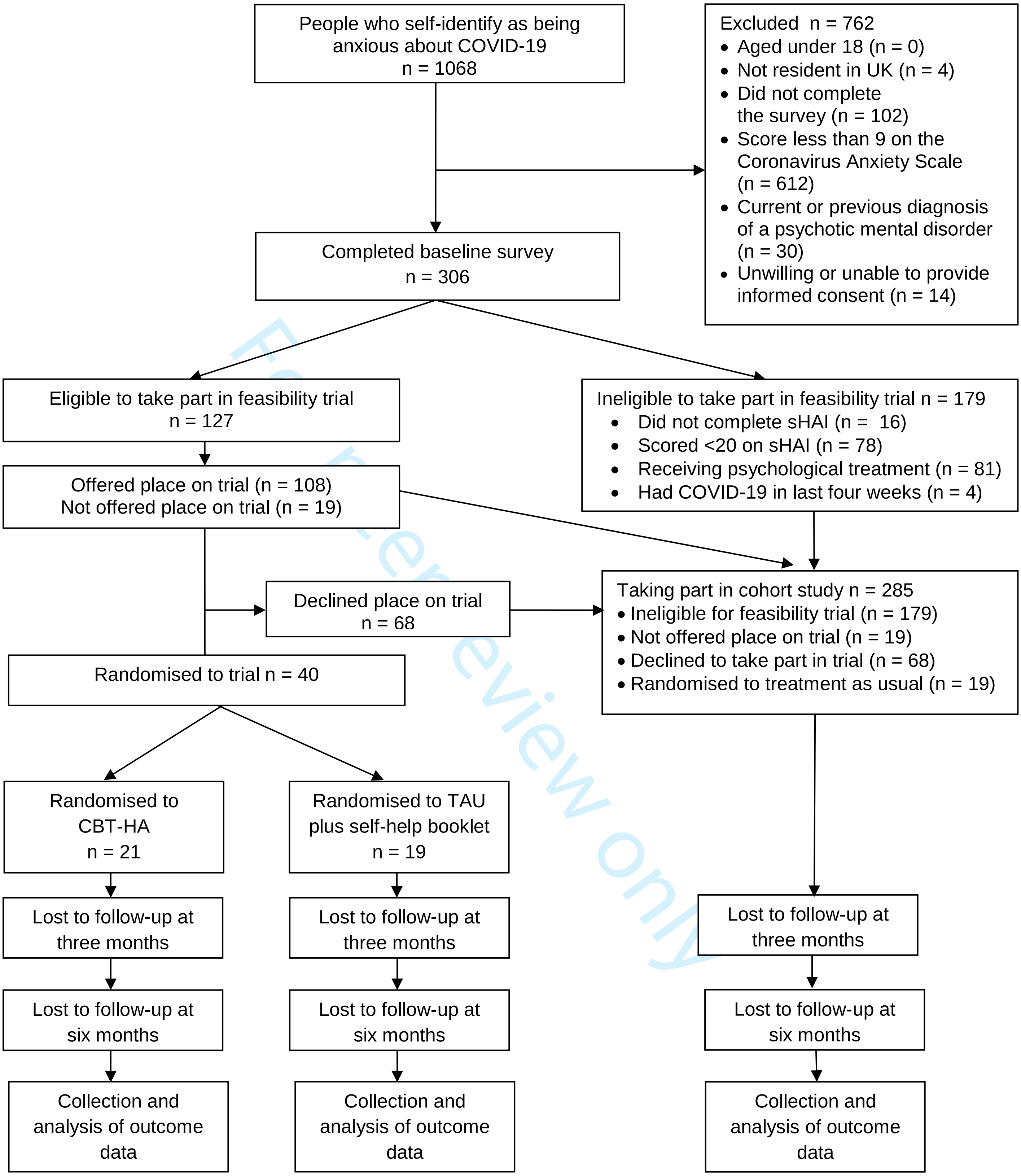

CBT-HA = Cognitive Behaviour Therapy for health anxiety. sHAI = Short for of Health Anxiety Inventory. TAU = Treatment as usual

Figure 1. Study flow diagram

For peer review only - http://bmjopen.bmj.com/site/about/guidelines.xhtml

Imperial College London

Chief Investigator: Prof. Mike Crawford
Division of Psychiatry, Imperial College London
7th Floor Commonwealth Building, Du Cane Road
London. W12 0NN

Consent Form for the COVID Anxiety Project

This consent form should be completed after reading the COVID Anxiety Project Information Sheet version 1.0, date 09.01.2021

Please sign your initials in each box to confirm the statements

I am 18 years or older.	
I have read and understand the information sheet for the COVID Anxiety Project. I have had the opportunity to consider the information, ask questions and any questions were answered satisfactorily.	
I understand that my participation is voluntary and that I am free to withdraw at any time without giving any reason, without my medical care or legal rights being affected.	
I agree for my data to be recorded and stored on the Qualtrics server in compliance with the General Data Proection Regulations (GDPR). I agree to Imperial College keeping my data and using it for the purpose of this research. I understand that the responses that I give will be anonymised and kept for 10 years.	
I understand that my contact details will be kept by Imperial College until then end of the project and then deleted.	
I understand that I will be offered a £30 voucher when I have completed the six month questionnaire.	
I agree to take part in the COVID Anxiety Project.	

For peer review only

Please write your full name, today's date and add your signature below

Name of individual _______________ Date _______________ Signature _______________

For peer review only - http://bmjopen.bmj.com/site/about/guidelines.xhtml

For peer review only

For peer review only - http://bmjopen.bmj.com/site/about/guidelines.xhtml

Imperial College London

Chief Investigator: Prof. Mike Crawford
Division of Psychiatry, Imperial College London
7th Floor Commonwealth Building, Du Cane Road
London. W12 0NN

Consent Form for the COVID Anxiety Project Separate consent for Clinical Trial of CBT for Health Anxiety

This consent form should be completed after reading the COVID Anxiety Project: Clinical Trial Information Sheet

Please sign your initials in each box to confirm the statements

I confirm that I have read and understand the information sheet for the above study. I have had the opportunity to ask questions which have been answered fully	
I understand that by taking part in the trial, I have an equal chance of being offered therapy or no therapy. If I allocated to the group that receives therapy, I will be asked to take part in a series of one-to-one telephone sessions of cognitive behavioural therapy.	
I understand that my participation is voluntary and that I am free to withdraw at any time without giving any reason, without my medical care or legal rights being affected.	
I agree to Imperial College keeping my data and using it for the purpose of this research. I understand that the responses that I give will be anonymised and kept for 10 years.	
I understand that therapy sessions will be audio recorded so that a senior member of the research team may listen to the session and provide feedback to the therapist. The recordings wil be deleted at the end of the study.	
I consent to take part in the above study.	

Please write your full name, today's date and add your signature below

Name ______________________ Date ______________ Signature ______________________

For peer review only - http://bmjopen.bmj.com/site/about/guidelines.xhtml

STANDARD PROTOCOL ITEMS: RECOMMENDATIONS FOR INTERVENTIONAL TRIALS

SPIRIT 2013 Checklist: Recommended items to address in a clinical trial protocol and related documents*

Section/item	Item No	Description	Page number
**Administrative information**			
Title	1	Descriptive title identifying the study design, population, interventions, and, if applicable, trial acronym	1
Trial registration	2a	Trial identifier and registry name. If not yet registered, name of intended registry	2
	2b	All items from the World Health Organization Trial Registration Data Set	throughout
Protocol version	3	Date and version identifier	
Funding	4	Sources and types of financial, material, and other support	14
Roles and responsibilities	5a	Names, affiliations, and roles of protocol contributors	14
	5b	Name and contact information for the trial sponsor	5
	5c	Role of study sponsor and funders, if any, in study design; collection, management, analysis, and interpretation of data; writing of the report; and the decision to submit the report for publication, including whether they will have ultimate authority over any of these activities	14
	5d	Composition, roles, and responsibilities of the coordinating centre, steering committee, endpoint adjudication committee, data management team, and other individuals or groups overseeing the trial, if applicable (see Item 21a for data monitoring committee)	14
**Introduction**			
Background and rationale	6a	Description of research question and justification for undertaking the trial, including summary of relevant studies (published and unpublished) examining benefits and harms for each intervention	3-4
	6b	Explanation for choice of comparators	9-10
Objectives	7	Specific objectives or hypotheses	4

For peer review only

Trial design	8	Description of trial design including type of trial (eg, parallel group, crossover, factorial, single group), allocation ratio, and framework (eg, superiority, equivalence, noninferiority, exploratory)	4
**Methods: Participants, interventions, and outcomes**			
Study setting	9	Description of study settings (eg, community clinic, academic hospital) and list of countries where data will be collected. Reference to where list of study sites can be obtained	5
Eligibility criteria	10	Inclusion and exclusion criteria for participants. If applicable, eligibility criteria for study centres and individuals who will perform the interventions (eg, surgeons, psychotherapists)	5
Interventions	11a	Interventions for each group with sufficient detail to allow replication, including how and when they will be administered	9-10
	11b	Criteria for discontinuing or modifying allocated interventions for a given trial participant (eg, drug dose change in response to harms, participant request, or improving/worsening disease)	Not applicable
	11c	Strategies to improve adherence to intervention protocols, and any procedures for monitoring adherence (eg, drug tablet return, laboratory tests)	Not applicable
	11d	Relevant concomitant care and interventions that are permitted or prohibited during the trial	9
Outcomes	12	Primary, secondary, and other outcomes, including the specific measurement variable (eg, systolic blood pressure), analysis metric (eg, change from baseline, final value, time to event), method of aggregation (eg, median, proportion), and time point for each outcome. Explanation of the clinical relevance of chosen efficacy and harm outcomes is strongly recommended	7-8, 11
Participant timeline	13	Time schedule of enrolment, interventions (including any run-ins and washouts), assessments, and visits for participants. A schematic diagram is highly recommended (see Figure)	20
Sample size	14	Estimated number of participants needed to achieve study objectives and how it was determined, including clinical and statistical assumptions supporting any sample size calculations	10

Recruitment	15	Strategies for achieving adequate participant enrolment to reach target sample size	5-6
**Methods: Assignment of interventions (for controlled trials)**			
Allocation:			
Sequence generation	16a	Method of generating the allocation sequence (eg, computer-generated random numbers), and list of any factors for stratification. To reduce predictability of a random sequence, details of any planned restriction (eg, blocking) should be provided in a separate document that is unavailable to those who enrol participants or assign interventions	7
Allocation concealment mechanism	16b	Mechanism of implementing the allocation sequence (eg, central telephone; sequentially numbered, opaque, sealed envelopes), describing any steps to conceal the sequence until interventions are assigned	7
Implementatio n	16c	Who will generate the allocation sequence, who will enrol participants, and who will assign participants to interventions	7
Blinding (masking)	17a	Who will be blinded after assignment to interventions (eg, trial participants, care providers, outcome assessors, data analysts), and how	6-7
	17b	If blinded, circumstances under which unblinding is permissible, and procedure for revealing a participant's allocated intervention during the trial	Not applicable
**Methods: Data collection, management, and analysis**			
Data collection methods	18a	Plans for assessment and collection of outcome, baseline, and other trial data, including any related processes to promote data quality (eg, duplicate measurements, training of assessors) and a description of study instruments (eg, questionnaires, laboratory tests) along with their reliability and validity, if known. Reference to where data collection forms can be found, if not in the protocol	8
	18b	Plans to promote participant retention and complete follow-up, including list of any outcome data to be collected for participants who discontinue or deviate from intervention protocols	8

Data management	19	Plans for data entry, coding, security, and storage, including any related processes to promote data quality (eg, double data entry; range checks for data values). Reference to where details of data management procedures can be found, if not in the protocol	12-13
Statistical methods	20a	Statistical methods for analysing primary and secondary outcomes. Reference to where other details of the statistical analysis plan can be found, if not in the protocol	10-11
	20b	Methods for any additional analyses (eg, subgroup and adjusted analyses)	11
	20c	Definition of analysis population relating to protocol non-adherence (eg, as randomised analysis), and any statistical methods to handle missing data (eg, multiple imputation)	10-11
**Methods: Monitoring**			
Data monitoring	21a	Composition of data monitoring committee (DMC); summary of its role and reporting structure; statement of whether it is independent from the sponsor and competing interests; and reference to where further details about its charter can be found, if not in the protocol. Alternatively, an explanation of why a DMC is not needed	14
	21b	Description of any interim analyses and stopping guidelines, including who will have access to these interim results and make the final decision to terminate the trial	Not applicable
Harms	22	Plans for collecting, assessing, reporting, and managing solicited and spontaneously reported adverse events and other unintended effects of trial interventions or trial conduct	8-9
Auditing	23	Frequency and procedures for auditing trial conduct, if any, and whether the process will be independent from investigators and the sponsor	12
**Ethics and dissemination**			
Research ethics approval	24	Plans for seeking research ethics committee/institutional review board (REC/IRB) approval	1, 11-12

Protocol amendments	25	Plans for communicating important protocol modifications (eg, changes to eligibility criteria, outcomes, analyses) to relevant parties (eg, investigators, REC/IRBs, trial participants, trial registries, journals, regulators)	Not applicable
Consent or assent	26a	Who will obtain informed consent or assent from potential trial participants or authorised surrogates, and how (see Item 32)	11-12
	26b	Additional consent provisions for collection and use of participant data and biological specimens in ancillary studies, if applicable	Not applicable
Confidentiality	27	How personal information about potential and enrolled participants will be collected, shared, and maintained in order to protect confidentiality before, during, and after the trial	12
Declaration of interests	28	Financial and other competing interests for principal investigators for the overall trial and each study site	14
Access to data	29	Statement of who will have access to the final trial dataset, and disclosure of contractual agreements that limit such access for investigators	14
Ancillary and post-trial care	30	Provisions, if any, for ancillary and post-trial care, and for compensation to those who suffer harm from trial participation	Not applicable
Dissemination policy	31a	Plans for investigators and sponsor to communicate trial results to participants, healthcare professionals, the public, and other relevant groups (eg, via publication, reporting in results databases, or other data sharing arrangements), including any publication restrictions	12-13
	31b	Authorship eligibility guidelines and any intended use of professional writers	Not applicable
	31c	Plans, if any, for granting public access to the full protocol, participant-level dataset, and statistical code	Not applicable
**Appendices**			
Informed consent materials	32	Model consent form and other related documentation given to participants and authorised surrogates	Available on request
Biological specimens	33	Plans for collection, laboratory evaluation, and storage of biological specimens for genetic or molecular analysis in the current trial and for future use in ancillary studies, if applicable	Not applicable

For peer review only - http://bmjopen.bmj.com/site/about/guidelines.xhtml

*It is strongly recommended that this checklist be read in conjunction with the SPIRIT 2013 Explanation & Elaboration for important clarification on the items. Amendments to the protocol should be tracked and dated. The SPIRIT checklist is copyrighted by the SPIRIT Group under the Creative Commons "Attribution-NonCommercial-NoDerivs 3.0 Unported" license.

For peer review only
